# Supplementary material for: Sequence variant affects GCSAML splicing, mast cell specific proteins, and risk of urticaria
Source: Commun Biol. 2023 Jul 10;6:703. doi: 10.1038/s42003-023-05079-4 (PMC10333346; doi:10.1038/s42003-023-05079-4)
Supplement: Supplementary file 2 — Supplementary Information [file 42003_2023_5079_MOESM2_ESM.pdf]

Sequence variant affects *GCSAML* splicing, mast cell specific proteins, and risk of urticaria
Supplementary Figures

Ragnar P Kristjansson[1], Gudjon R Oskarsson[1,2], Astros Skuladottir[1], Asmundur Oddsson[1], Solvi Rognvaldsson[1], Gardar Sveinbjornsson[1], Sigrun H Lund[1], Brynjar O Jensson[1], Edda L Styrmisdottir[1], Gisli H Halldorsson[1], Egil Ferkingstad[1], Grimur Hjorleifsson Eldjarn[1], Doruk Beyter[1], Snædis Kristmundsdottir[1,3], Kristinn Juliusson[1], Run Fridriksdottir[1], Gudny A Arnadottir[1], Hildigunnur Katrinardottir[1], Margret H Snorradottir[1], Vinicius Tragante[1], Lilja Stefansdottir[1], Erna V. Ivarsdottir[1,4], Gyda Bjornsdottir[1], Bjarni V Halldorsson[1,3], Gudmar Thorleifsson[1], Bjorn R Ludviksson[2,5], Pall T Onundarson[2,6], Saedis Saevarsdottir[1,2,7,8], Pall Melsted[1,4], Gudmundur L Norddahl[1], Unnur S Bjornsdottir[8,9], Thorunn Olafsdottir[1,2], Daniel F Gudbjartsson[1,4], Unnur Thorsteinsdottir[1,2], Ingileif Jonsdottir[1,2], Patrick Sulem[1], Kari Stefansson[1,2]

1. deCODE genetics/Amgen Inc., Reykjavik, Iceland
2. Faculty of Medicine, School of Health Sciences, University of Iceland, Reykjavik, Iceland
3. School of Science and Engineering, Reykjavik University, Reykjavik, Iceland
4. School of Engineering and Natural Sciences, University of Iceland, Reykjavik, Iceland
5. Department of Immunology, Landspitali, the National University Hospital of Iceland, Reykjavik, Iceland
6. Department of Laboratory Hematology, Landspitali, the National University Hospital of Iceland, Reykjavik, Iceland
7. Rheumatology Unit, Department of Medicine, Karolinska Institutet, Solna, Stockholm, Sweden.
8. Department of Medicine, Landspitali, the National University Hospital of Iceland, Reykjavik, Iceland
9. The Medical Center Mjodd, Reykjavik, Iceland

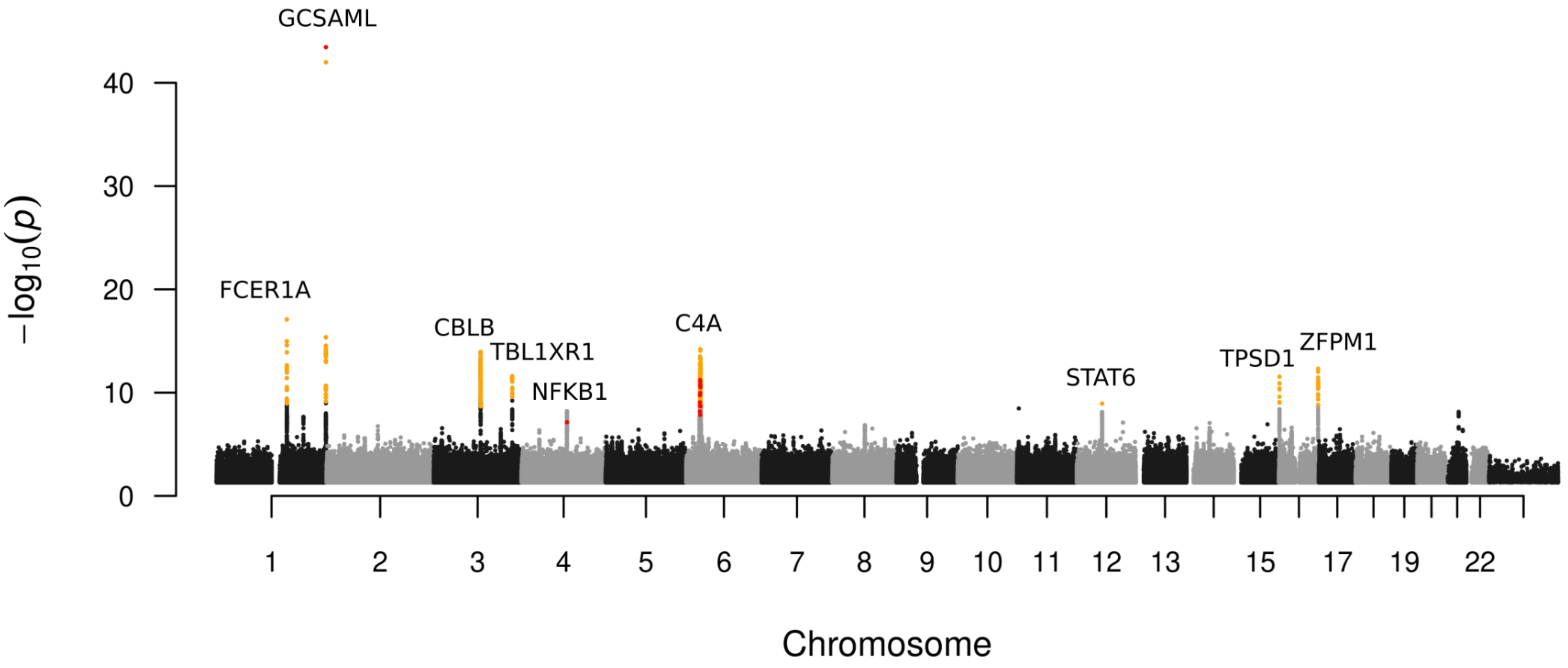

Supplementary Figure 1: Manhattan plot for the meta-analysis of GWAS on urticaria from Iceland, the UK, Finnland, and Japan (n = 40,694 cases and 1,230,001 controls). Nine loci harbor genome-wide significant signals. All variants with P values below their respective variant-class thresholds are depicted yellow. Predicted protein altering sequence variants are colored red. Variants are plotted by chromosomal position (x axis) and $-\log_{10}$[P] values (y axis; two-sided loistic regression). For clarity, only variants with $P < 0.05$ are plotted.

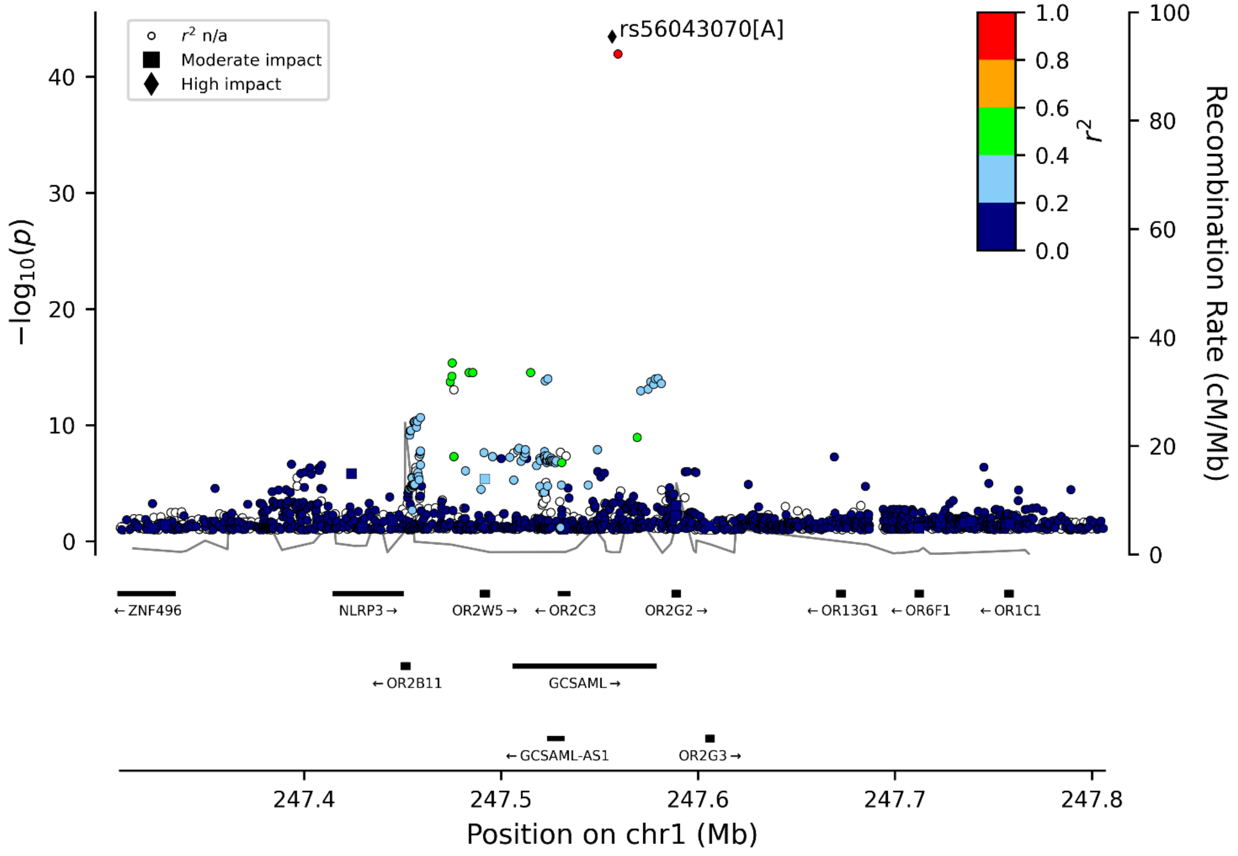

Supplementary Figure 2: Locus plot showing the associations of variants at the *GCSAML* locus associated with urticaria under the additive model in a meta-analysis of GWAS from Iceland, the UK, Finland, and Japan (n = 40,694 cases and 1,230,001 controls). The leading variant is indicated by its rs number, and other variants are colored according to correlation (r^2) with the leading marker (legend at top-right). Correlation between variants is estimated using genotype data from the Icelandic population. $-\log_{10}P$ values are shown along the left y-axis (two-sided logistic regression), and correspond to the variants depicted in the plot. The right y-axis shows calculated recombination rates at the chromosomal location, plotted as a solid black line. The leading variant is strongly correlated with the intronic rs74227709 in *GCSAML* (r^2=1.00, D'=1).

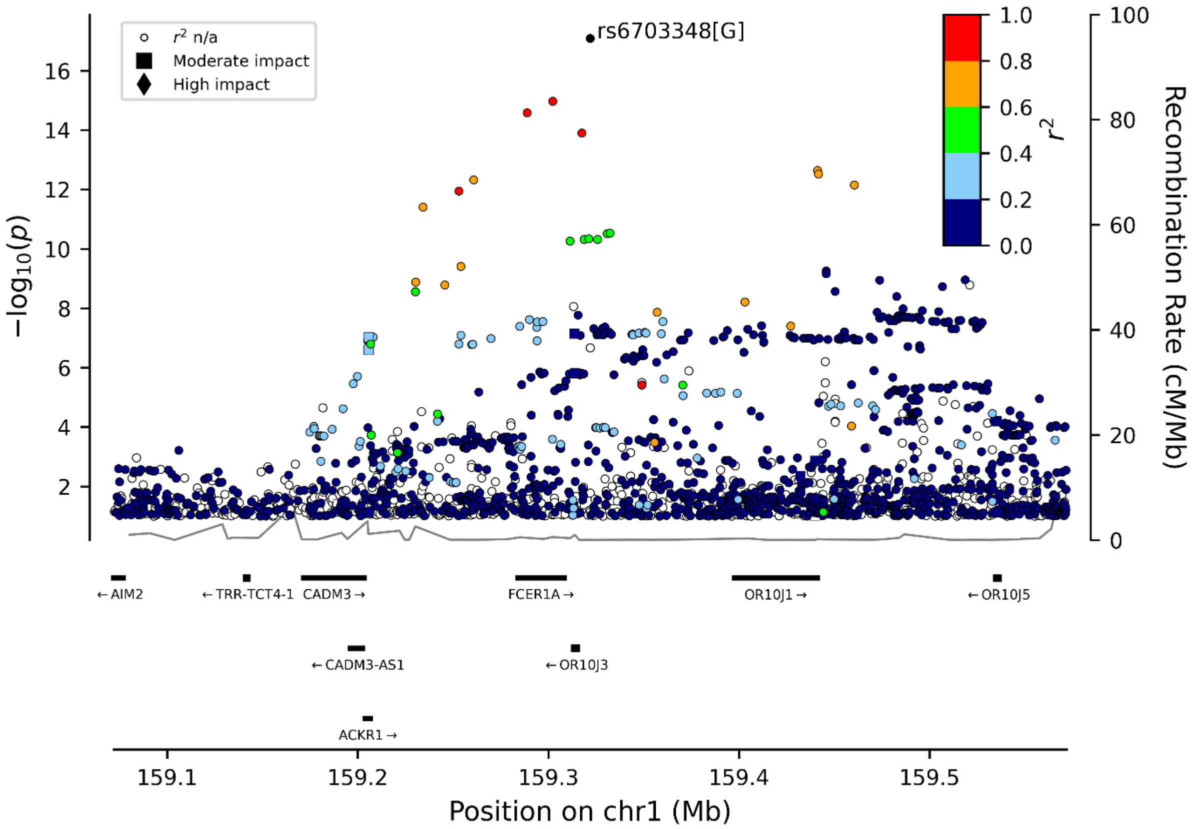

Supplementary Figure 3: Locus plot showing the associations of variants at the *FCER1A* locus associated with urticaria under the additive model in a meta-analysis of GWAS from Iceland, the UK, Finland, and Japan (n = 40,694 cases and 1,230,001 controls). The leading variant is indicated by its rs number, and other variants are colored according to correlation (r^2) with the leading marker (legend at top-right). Correlation between variants is estimated using genotype data from the Icelandic population. –log10P values are shown along the left y-axis (two-sided logistic regression), and correspond to the variants depicted in the plot. The right y-axis shows calculated recombination rates at the chromosomal location, plotted as a solid black line.

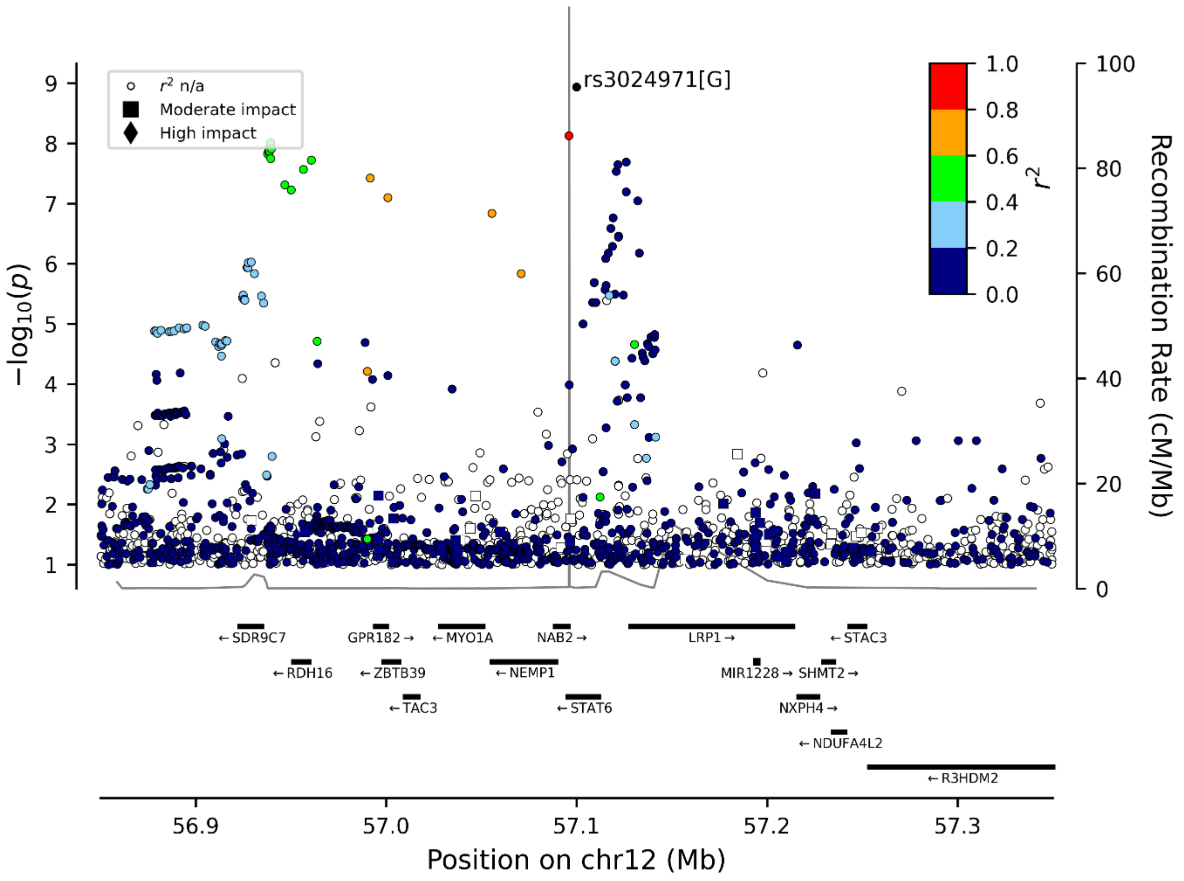

Supplementary Figure 4: Locus plot showing the associations of variants at the *STAT6* locus associated with urticaria under the additive model in a meta-analysis of GWAS from Iceland, the UK, Finland, and Japan (n = 40,694 cases and 1,230,001 controls). The leading variant is indicated by its rs number, and other variants are colored according to correlation (r^2) with the leading marker (legend at top-right). Correlation between variants is estimated using genotype data from the Icelandic population. –log10P values are shown along the left y-axis (two-sided logistic regression), and correspond to the variants depicted in the plot. The right y-axis shows calculated recombination rates at the chromosomal location, plotted as a solid black line.

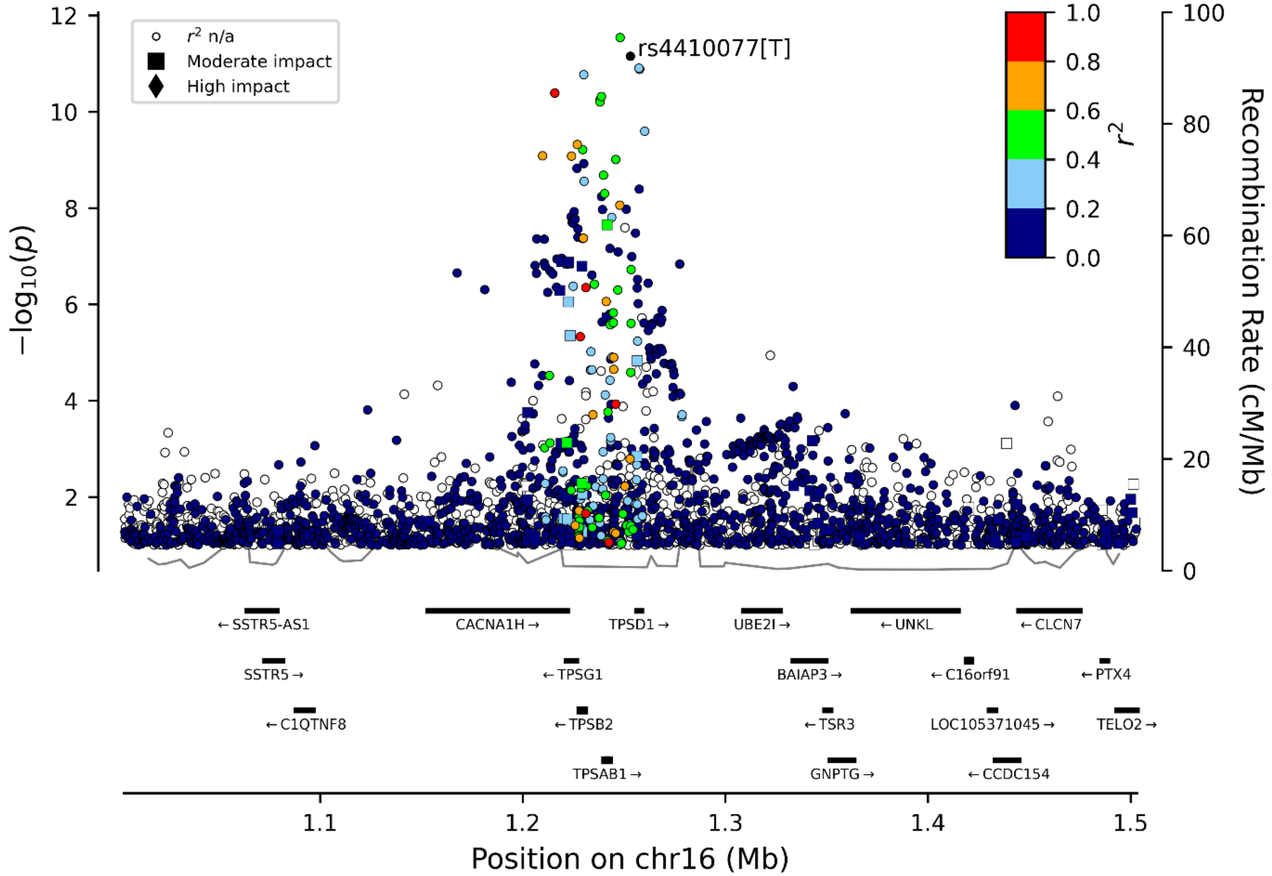

Supplementary Figure 5: Locus plot showing the associations of variants at the tryptase locus (rs4410077[T] is at *TPSD1*) associated with urticaria under the additive model in a meta-analysis of GWAS from Iceland, the UK, Finland, and Japan (n = 40,694 cases and 1,230,001 controls). The leading variant is indicated by its rs number, and other variants are colored according to correlation (r^2) with the leading marker (legend at top-right). Correlation between variants is estimated using genotype data from the Icelandic population. –log10P values are shown along the left y-axis (two-sided logistic regression), and correspond to the variants depicted in the plot. The right y-axis shows calculated recombination rates at the chromosomal location, plotted as a solid black line.

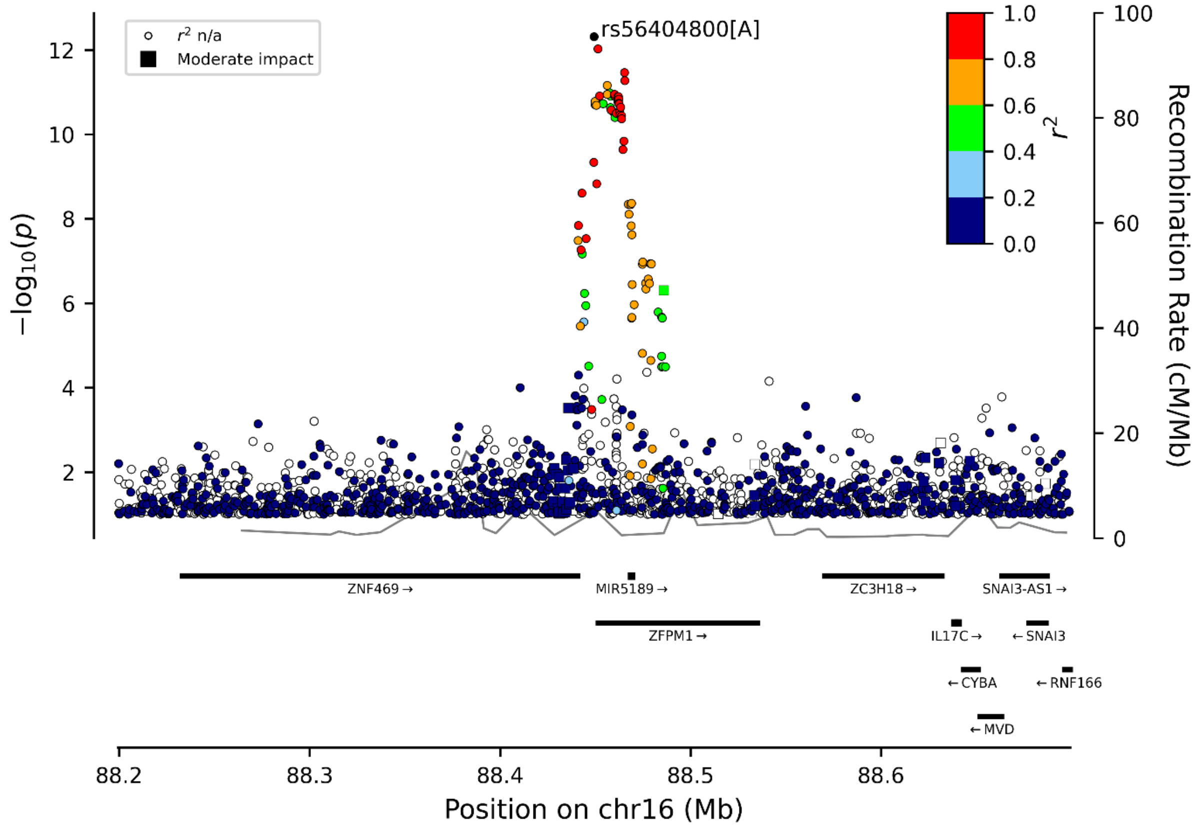

Supplementary Figure 6: Locus plot showing the associations of variants at the *ZFPM1* locus associated with urticaria under the additive model in a meta-analysis of GWAS from Iceland, the UK, Finland, and Japan (n = 40,694 cases and 1,230,001 controls). The leading variant is indicated by its rs number, and other variants are colored according to correlation (r^2) with the leading marker (legend at top-right). Correlation between variants is estimated using genotype data from the Icelandic population. –log10P values are shown along the left y-axis (two-sided logistic regression), and correspond to the variants depicted in the plot. The right y-axis shows calculated recombination rates at the chromosomal location, plotted as a solid black line.

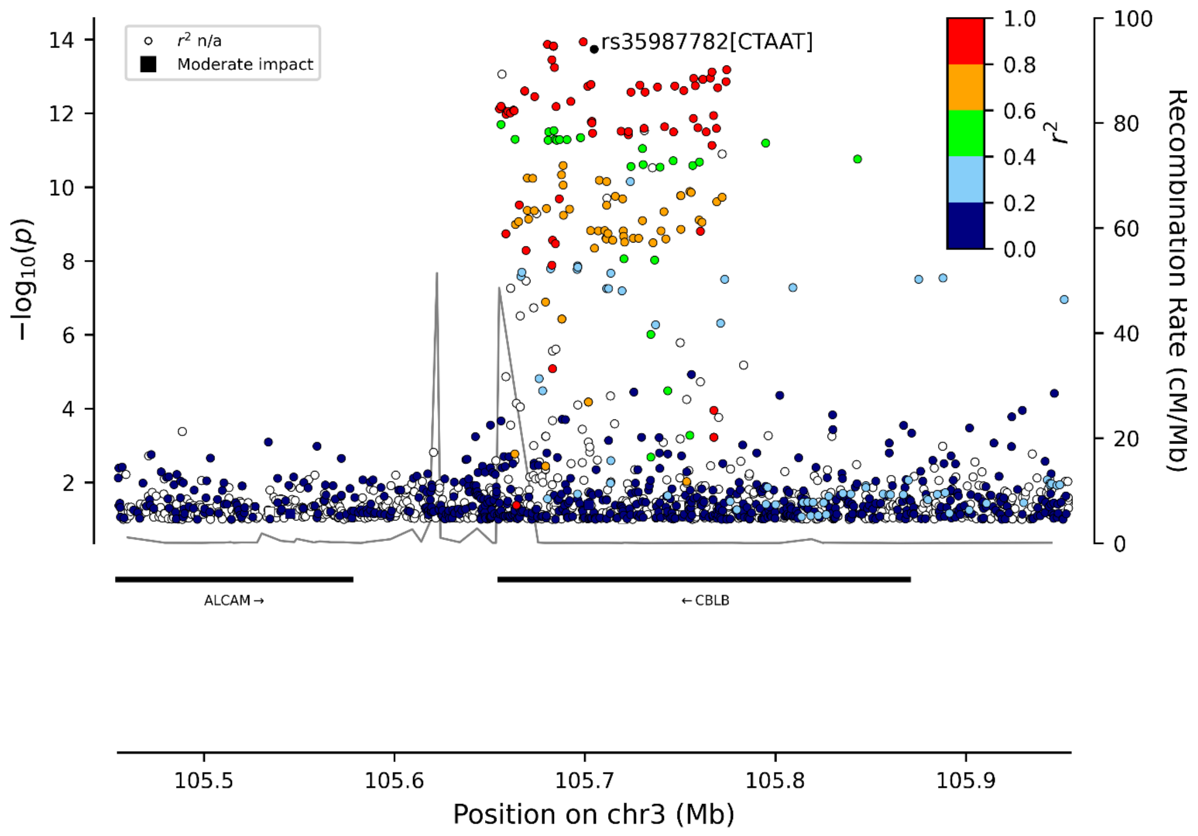

Supplementary Figure 7: Locus plot showing the associations of variants at the *CBLB* locus associated with urticaria under the additive model in a meta-analysis of GWAS from Iceland, the UK, Finland, and Japan (n = 40,694 cases and 1,230,001 controls). The leading variant is indicated by its rs number, and other variants are colored according to correlation (r^2) with the leading marker (legend at top-right). Correlation between variants is estimated using genotype data from the Icelandic population. –log10P values are shown along the left y-axis (two-sided logistic regression), and correspond to the variants depicted in the plot. The right y-axis shows calculated recombination rates at the chromosomal location, plotted as a solid black line.

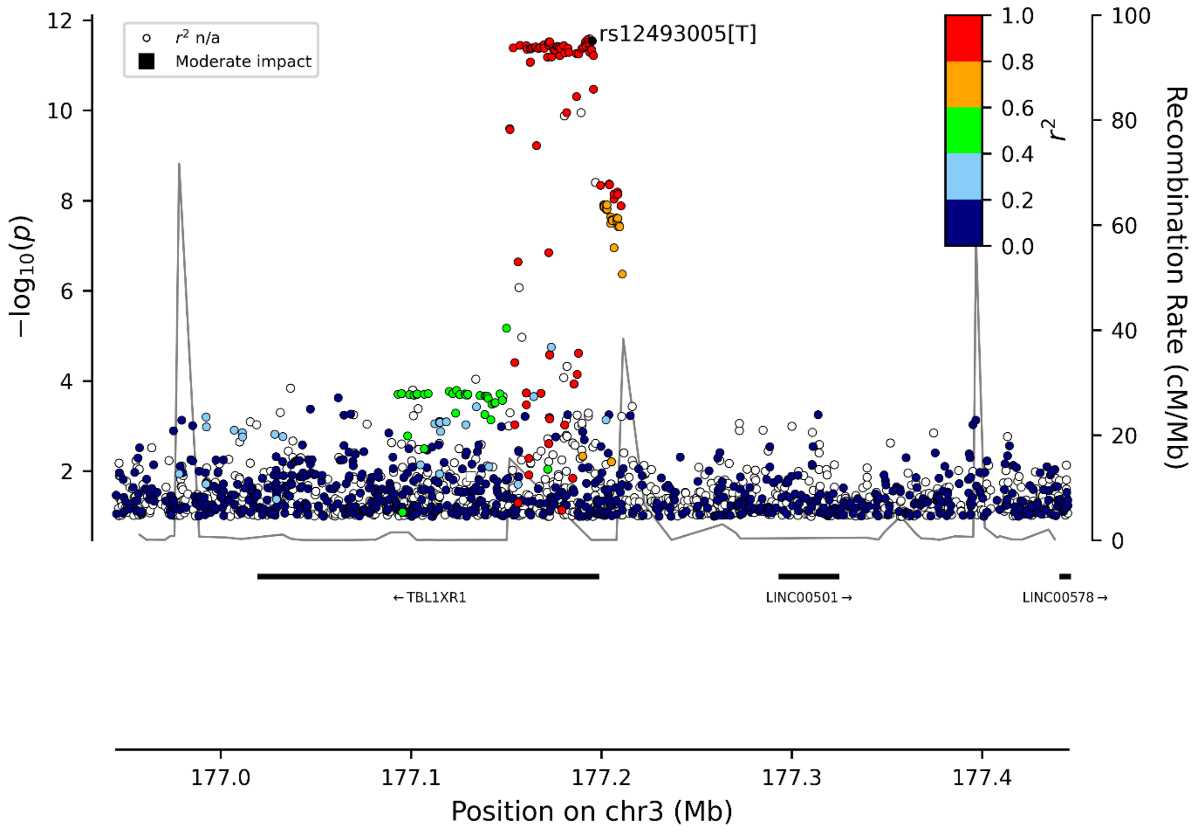

Supplementary Figure 8: Locus plot showing the associations of variants at the *TBL1XR1* locus associated with urticaria under the additive model in a meta-analysis of GWAS from Iceland, the UK, Finland, and Japan (n = 40,694 cases and 1,230,001 controls). The leading variant is indicated by its rs number, and other variants are colored according to correlation (r^2) with the leading marker (legend at top-right). Correlation between variants is estimated using genotype data from the Icelandic population. –log10P values are shown along the left y-axis (two-sided logistic regression), and correspond to the variants depicted in the plot. The right y-axis shows calculated recombination rates at the chromosomal location, plotted as a solid black line.

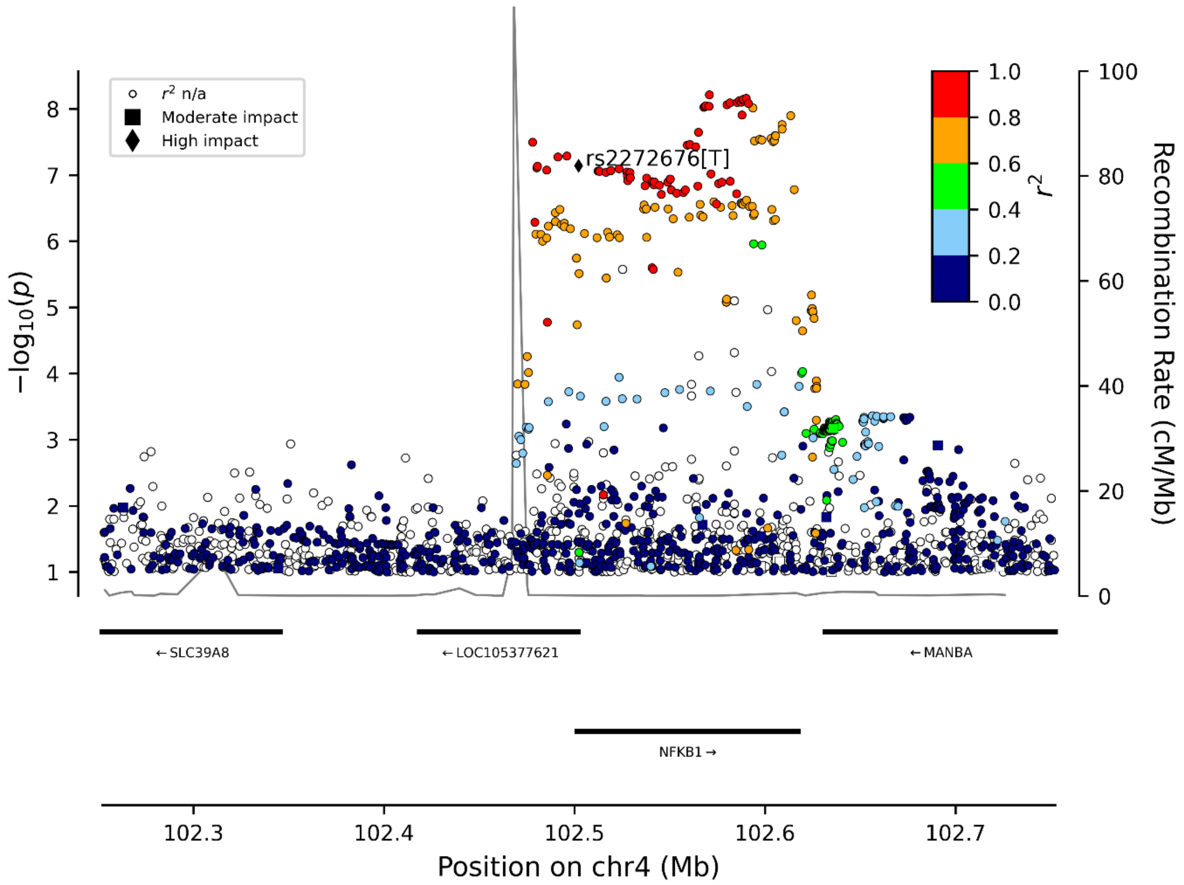

Supplementary Figure 9: Locus plot showing the associations of variants at the *NFKB1* locus associated with urticaria under the additive model in a meta-analysis of GWAS from Iceland, the UK, Finland, and Japan (n = 40,694 cases and 1,230,001 controls). The leading variant is indicated by its rs number, and other variants are colored according to correlation (r^2) with the leading marker (legend at top-right). Correlation between variants is estimated using genotype data from the Icelandic population. –log10P values are shown along the left y-axis (two-sided logistic regression), and correspond to the variants depicted in the plot. The right y-axis shows calculated recombination rates at the chromosomal location, plotted as a solid black line.

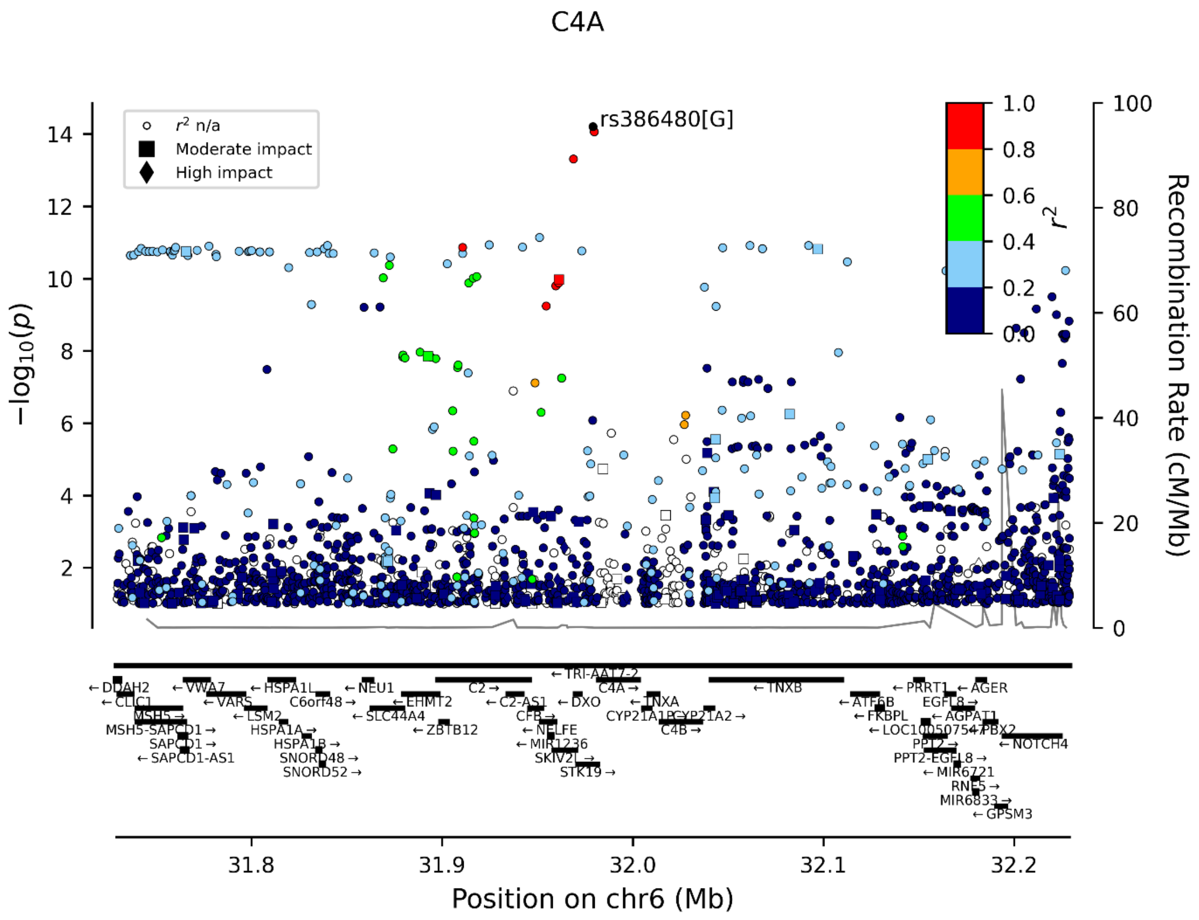

Supplementary Figure 10: Locus plot showing the associations of variants at the *C4A* locus associated with urticaria under the additive model in a meta-analysis of GWAS from Iceland, the UK, Finland, and Japan (n = 40,694 cases and 1,230,001 controls). The leading variant is indicated by its rs number, and other variants are colored according to correlation (r^2) with the leading marker (legend at top-right). Correlation between variants is estimated using genotype data from the Icelandic population. –log10P values are shown along the left y-axis (two-sided logistic regression), and correspond to the variants depicted in the plot. The right y-axis shows calculated recombination rates at the chromosomal location, plotted as a solid black line.

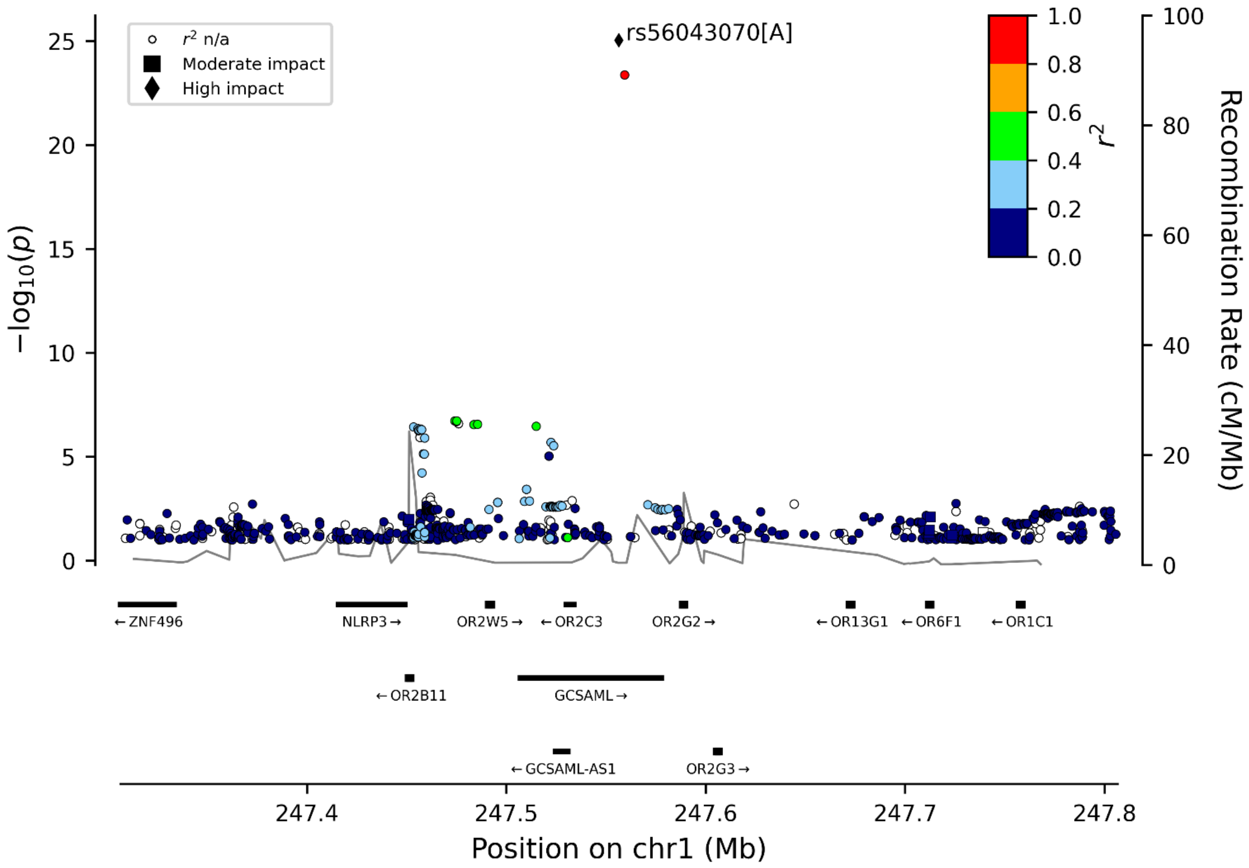

Supplementary Figure 11: Locus plot showing the associations of variants at the *GCSAML* locus associated with basophil percentage under the recessive model in a meta-analysis of GWAS from Iceland and the UK (n = 665,329). The leading variant is indicated by its rs number, and other variants are colored according to correlation (r^2) with the leading marker (legend at top-right). Correlation between variants is estimated using genotype data from the Icelandic population. Correlation between variants is estimated using genotype data from the Icelandic population . -$\log_{10}$P values are shown along the left y-axis (two-sided logistic regression), and correspond to the variants depicted in the plot. The right y-axis shows calculated recombination rates at the chromosomal location, plotted as a solid black line. The leading variant is strongly correlated with the intronic rs74227709 in *GCSAML* (r^2=1.00, D'=1).

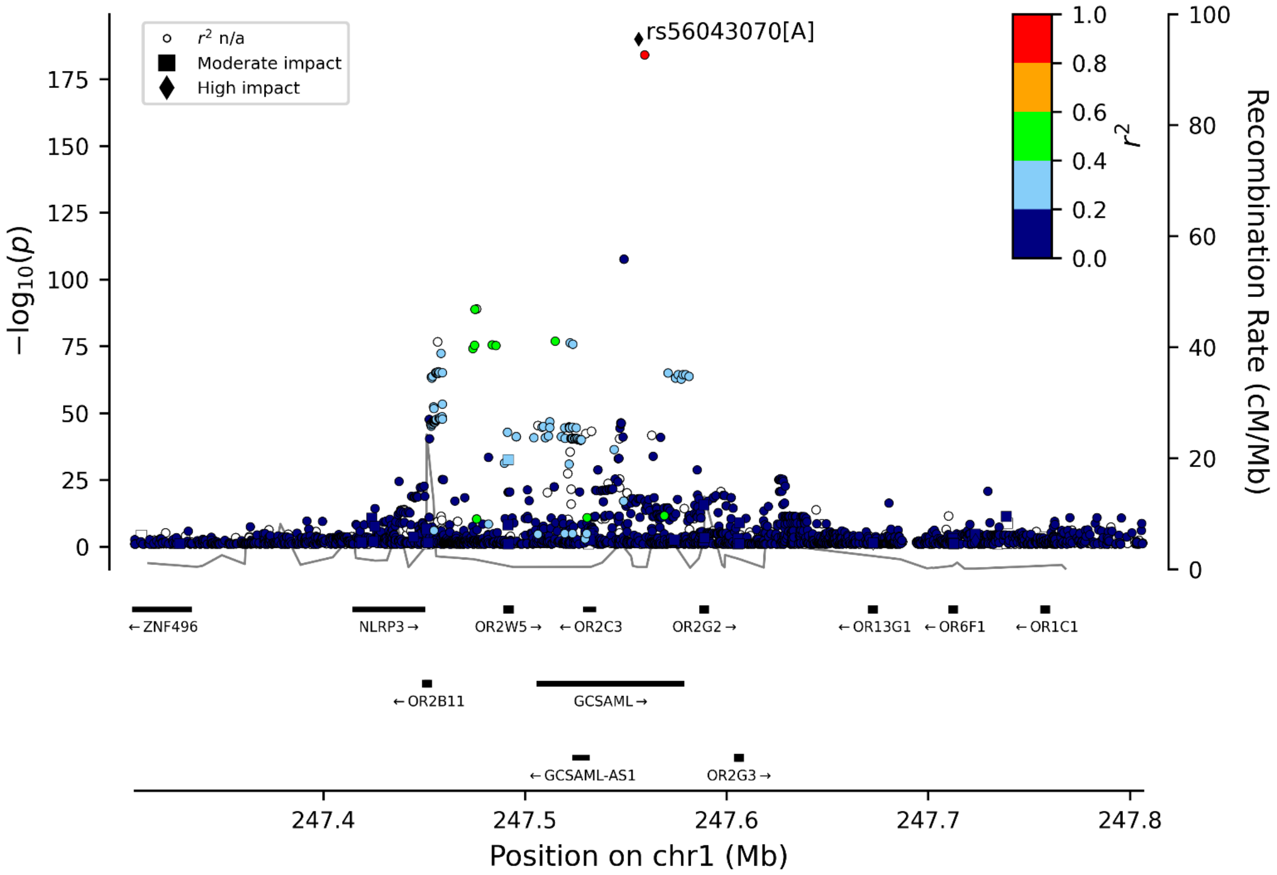

Supplementary Figure 12: : Locus plot showing the associations of variants at the *GCSAML* locus associated with platelet count under the additive model in a meta-analysis of GWAS from Iceland and the UK (n = 680,537). The leading variant is indicated by its rs number, and other variants are colored according to correlation (r^2) with the leading marker (legend at top-right). Correlation between variants is estimated using genotype data from the Icelandic population. Correlation between variants is estimated using genotype data from the Icelandic population . $-\log_{10}$P values are shown along the left y-axis (two-sided logistic regression), and correspond to the variants depicted in the plot. The right y-axis shows calculated recombination rates at the chromosomal location, plotted as a solid black line. The leading variant is strongly correlated with the intronic rs74227709 in *GCSAML* (r^2=1.00, D'=1).

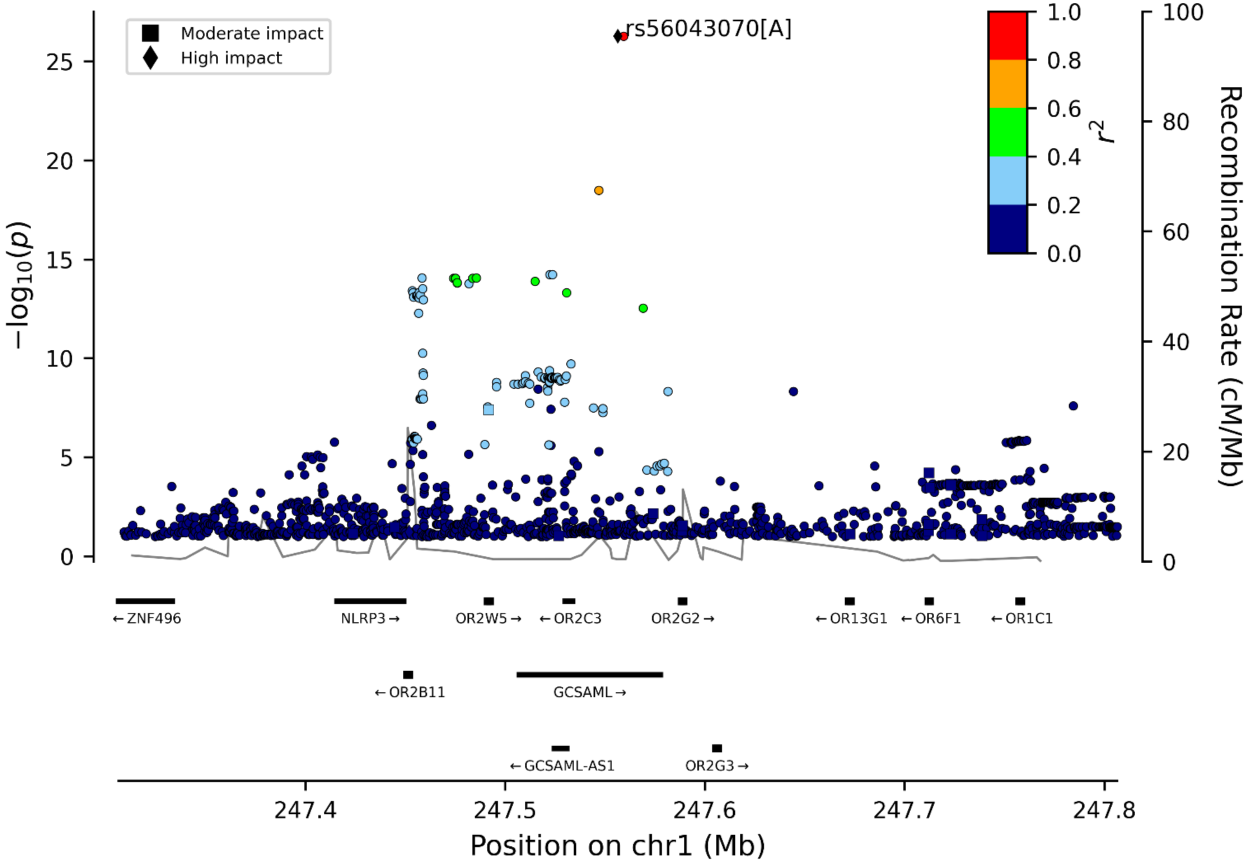

Supplementary Figure 13: Locus plots for the Icelandic data showing the associations of variants at the *GCSAML* locus with serum tryptase alpha/beta-1 (*TPSAB1*) levels under the additive model using the SomaLogic® SOMAscan platform (n = 35,559). The leading variant is indicated by its rs number, and other variants are colored according to correlation (r^2) with the leading marker (legend at top-right). Correlation between variants is estimated using genotype data from the Icelandic population. $-\log_{10}$P values are shown along the left y-axis (two-sided logistic regression), and correspond to the variants depicted in the plot. The right y-axis shows calculated recombination rates at the chromosomal location, plotted as a solid black line. The leading variant is fully correlated with the intronic rs74227709 in *GCSAML* (r^2=1.00, D'=1).).

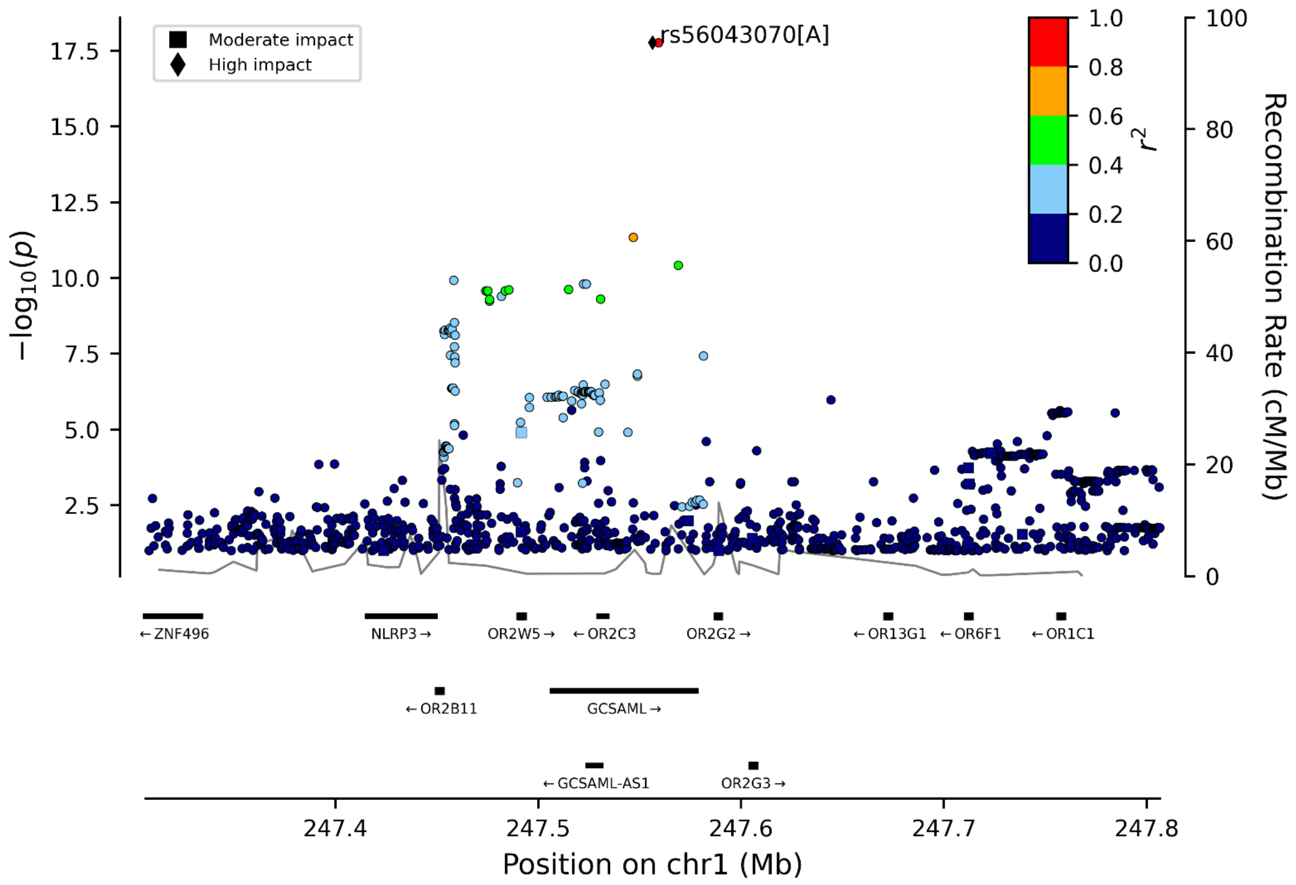

Supplementary Figure 14: Locus plots for the Icelandic data showing the associations of variants at the *GCSAML* locus with serum tryptase beta-2 (*TPSB2*) level under the additive model using the SomaLogic® SOMAscan platform (n = 35,559). The leading variant is indicated by its rs number, and other variants are colored according to correlation (r^2) with the leading marker (legend at top-right). Correlation between variants is estimated using genotype data from the Icelandic population. $-\log_{10}$P values are shown along the left y-axis (two-sided logistic regression), and correspond to the variants depicted in the plot. The right y-axis shows calculated recombination rates at the chromosomal location, plotted as a solid black line. The leading variant is strongly correlated with the intronic rs74227709 in *GCSAML* (r^2=1.00, D'=1).

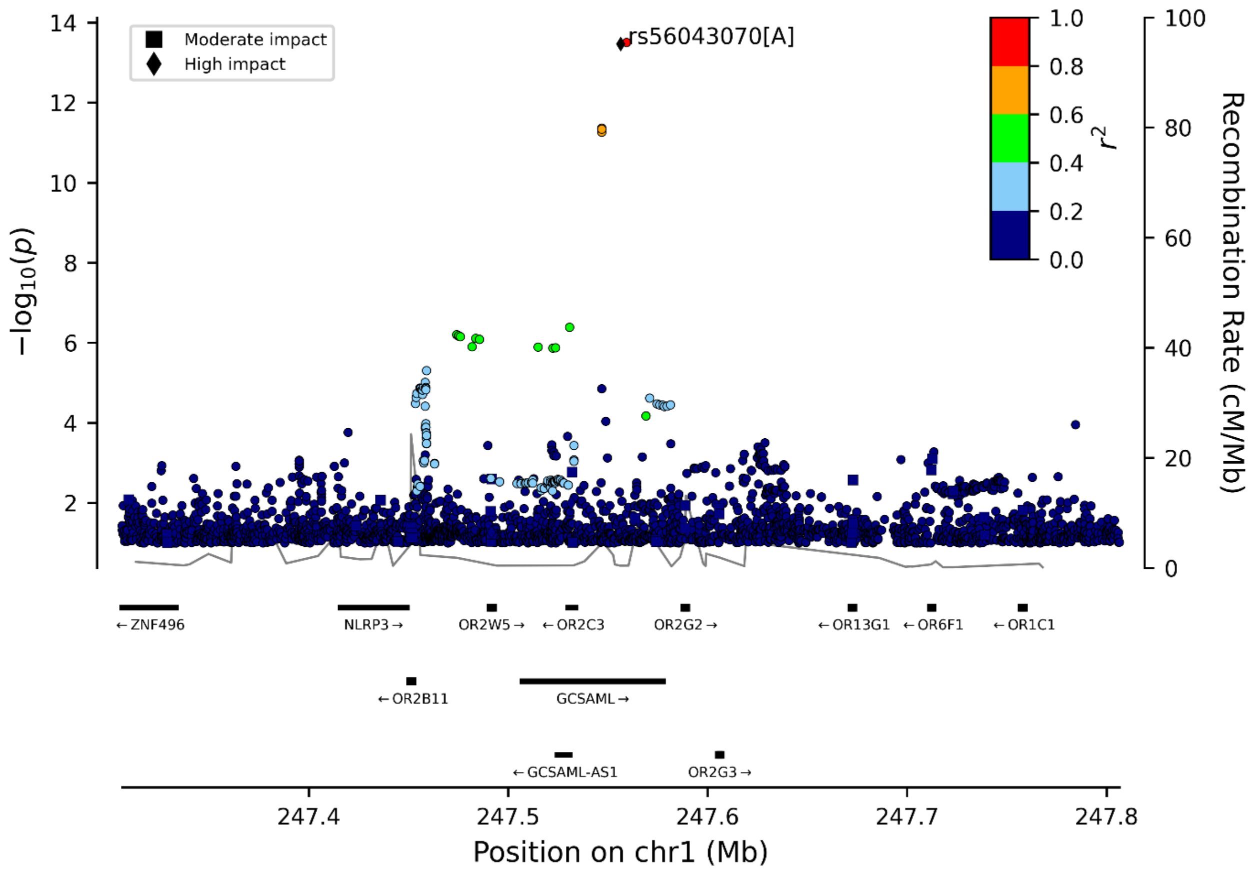

Supplementary Figure 15: Locus plot for the UK Biobank data showing the associations of variants at the *GCSAML* locus with serum serum tryptase alpha/beta-1 (*TPSAB1*) level under the additive model using the Olink Explore 3072 platform (n = 48,684). The leading variant is indicated by its rs number, and other variants are colored according to correlation (r^2) with the leading marker (legend at top-right). Correlation between variants is estimated using genotype data from the UK Biobank. –log10P values are shown along the left y-axis (two-sided logistic regression), and correspond to the variants depicted in the plot. The right y-axis shows calculated recombination rates at the chromosomal location, plotted as a solid black line. The leading variant is strongly correlated with the intronic rs74227709 in *GCSAML* (r^2=1.00, D'=1).

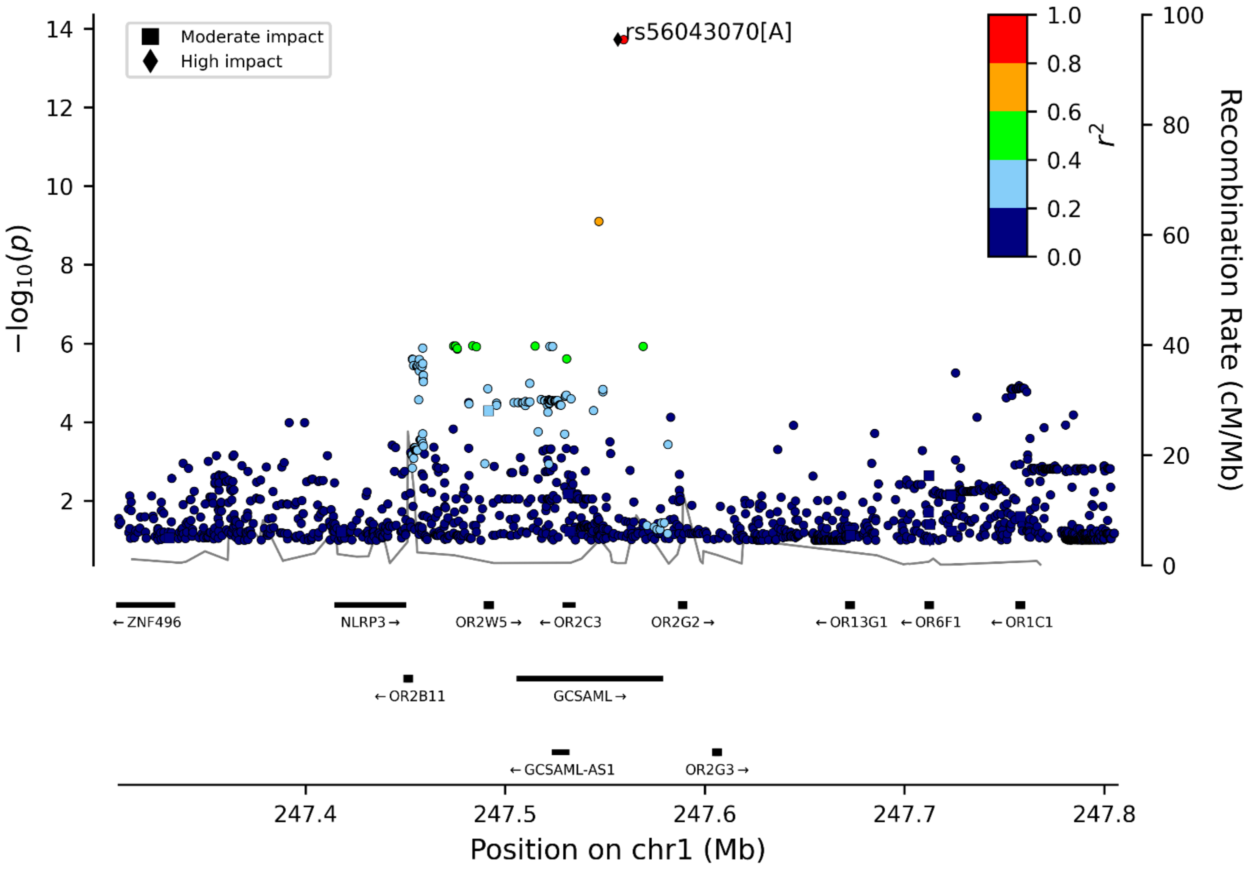

Supplementary Figure 16: Locus plots for the Icelandic data showing the associations of variants at the *GCSAML* locus with serum mast/stem cell factor receptor (SCFR) level (*KIT*) under the additive model using the SomaLogic® SOMAscan platform (n = 35,559). The leading variant is indicated by its rs number, and other variants are colored according to correlation (r^2) with the leading marker (legend at top-right). Correlation between variants is estimated using genotype data from the Icelandic population. $-\log_{10}P$ values are shown along the left y-axis (two-sided logistic regression), and correspond to the variants depicted in the plot. The right y-axis shows calculated recombination rates at the chromosomal location, plotted as a solid black line. The leading variant is strongly correlated with the intronic rs74227709 in *GCSAML* (r^2=1.00, D'=1).

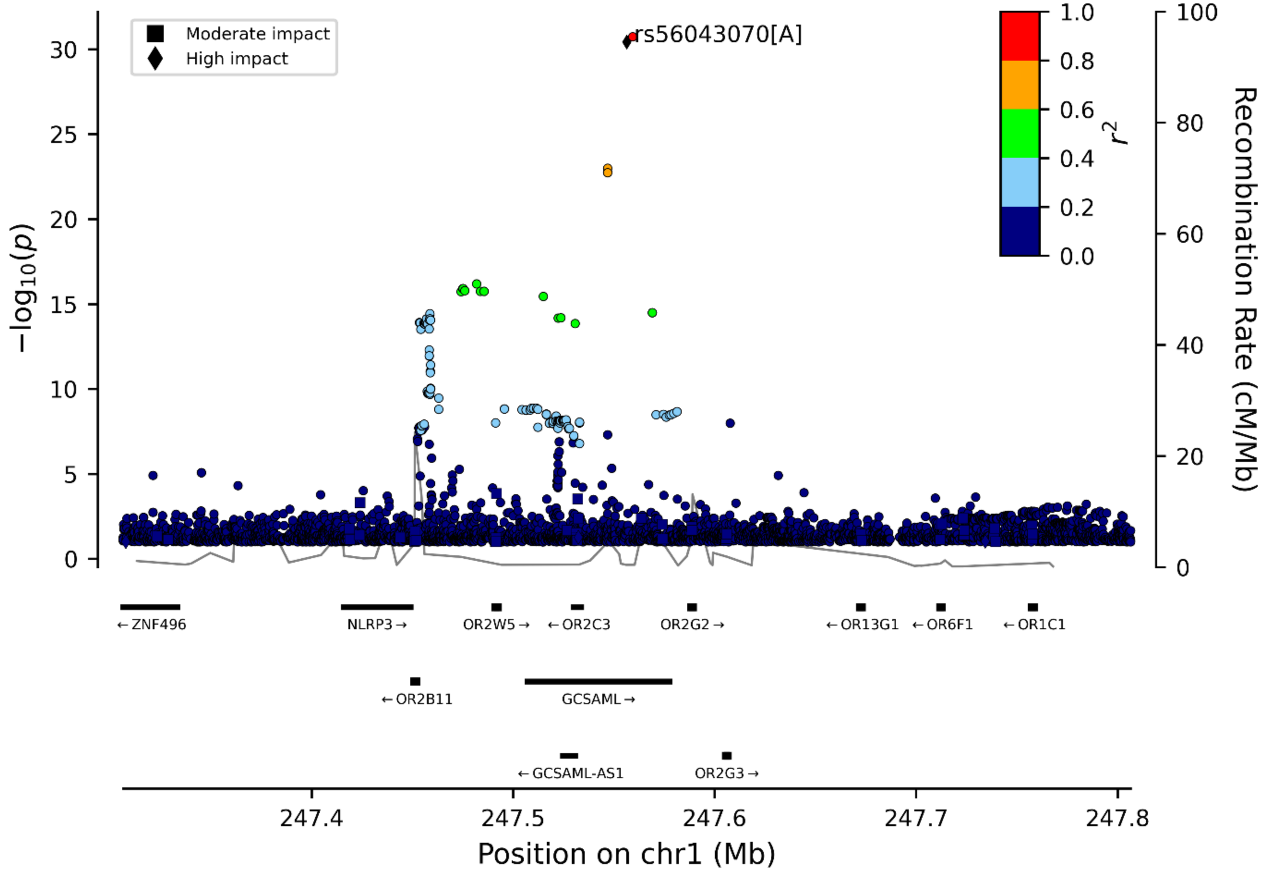

Supplementary Figure 17: Locus plot for the UK Biobank data showing the associations of variants at the *GCSAML* locus with serum mast/stem cell factor receptor (SCFR) level (*KIT*) under the additive model using the Olink Explore 3072 platform (n = 48,684). The leading variant is indicated by its rs number, and other variants are colored according to correlation (r^2) with the leading marker (legend at top-right). Correlation between variants is estimated using genotype data from the UK Biobank. –log10P values are shown along the left y-axis (two-sided logistic regression), and correspond to the variants depicted in the plot. The right y-axis shows calculated recombination rates at the chromosomal location, plotted as a solid black line. The leading variant is strongly correlated with the intronic rs74227709 in *GCSAML* (r^2=1.00, D'=1).

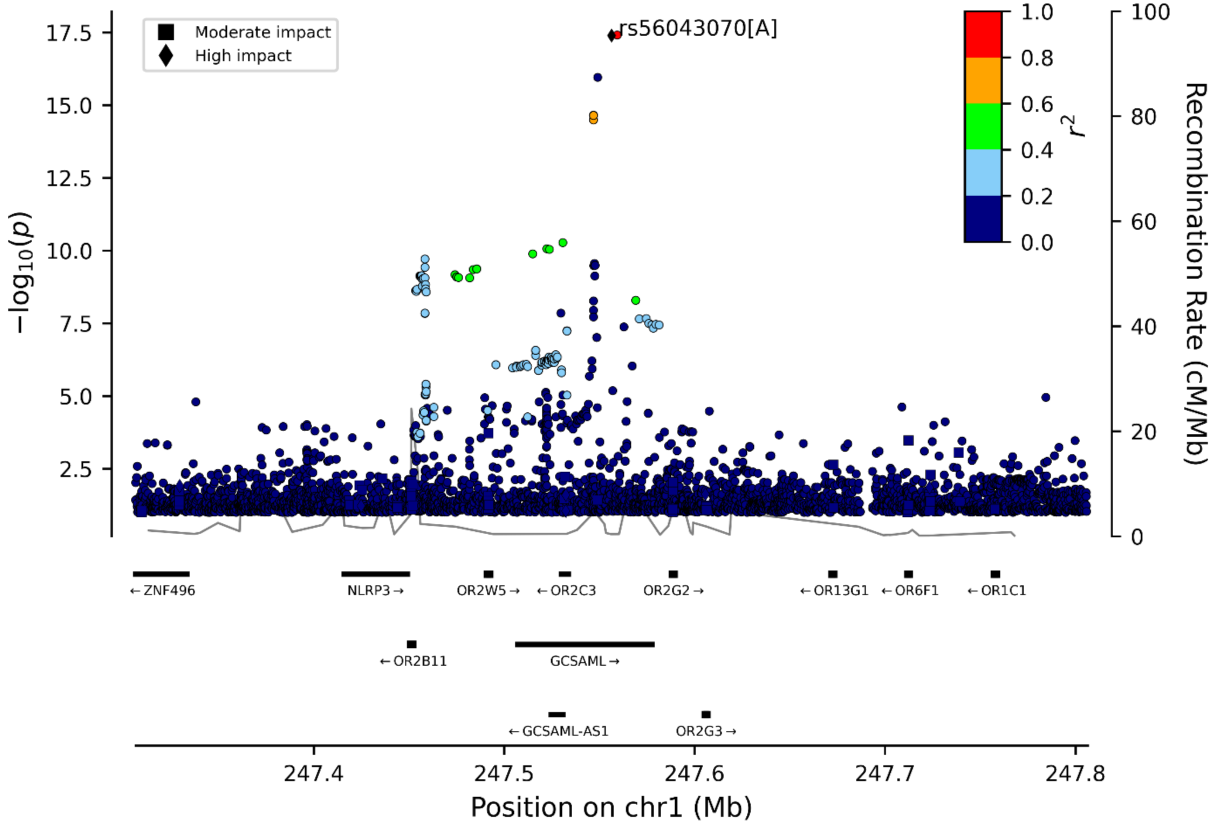

Supplementary Figure 18: Locus plot for the UK Biobank data showing the associations of variants at the *GCSAML* locus with P-selectin levels (*SELP*) under the additive model using the Olink Explore 3072 platform (n = 48,684). The leading variant is indicated by its rs number, and other variants are colored according to correlation (r^2) with the leading marker (legend at top-right). Correlation between variants is estimated using genotype data from the UK Biobank. –log10P values are shown along the left y-axis (two-sided logistic regression), and correspond to the variants depicted in the plot. The right y-axis shows calculated recombination rates at the chromosomal location, plotted as a solid black line. The leading variant is strongly correlated with the intronic rs74227709 in *GCSAML* (r^2=1.00, D'=1).

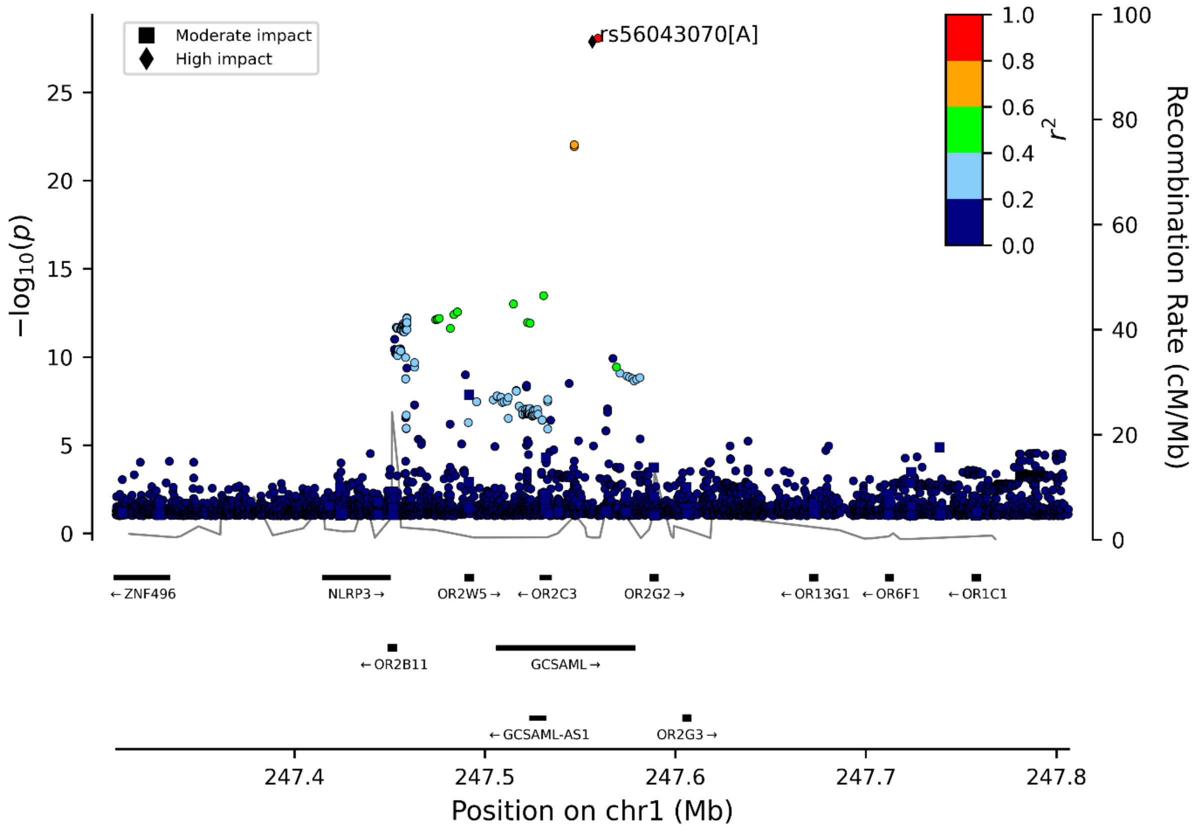

Supplementary Figure 19: Locus plot for the UK Biobank data showing the associations of variants at the *GCSAML* locus with sialic acid binding Ig like lectin 6 levels (*SIGLEC6*) under the additive model using the Olink Explore 3072 platform (n = 48,684). The leading variant is indicated by its rs number, and other variants are colored according to correlation (r^2) with the leading marker (legend at top-right). Correlation between variants is estimated using genotype data from the UK Biobank. –log10P values are shown along the left y-axis (two-sided logistic regression), and correspond to the variants depicted in the plot. The right y-axis shows calculated recombination rates at the chromosomal location, plotted as a solid black line. The leading variant is strongly correlated with the intronic rs74227709 in *GCSAML* (r^2=1.00, D'=1).

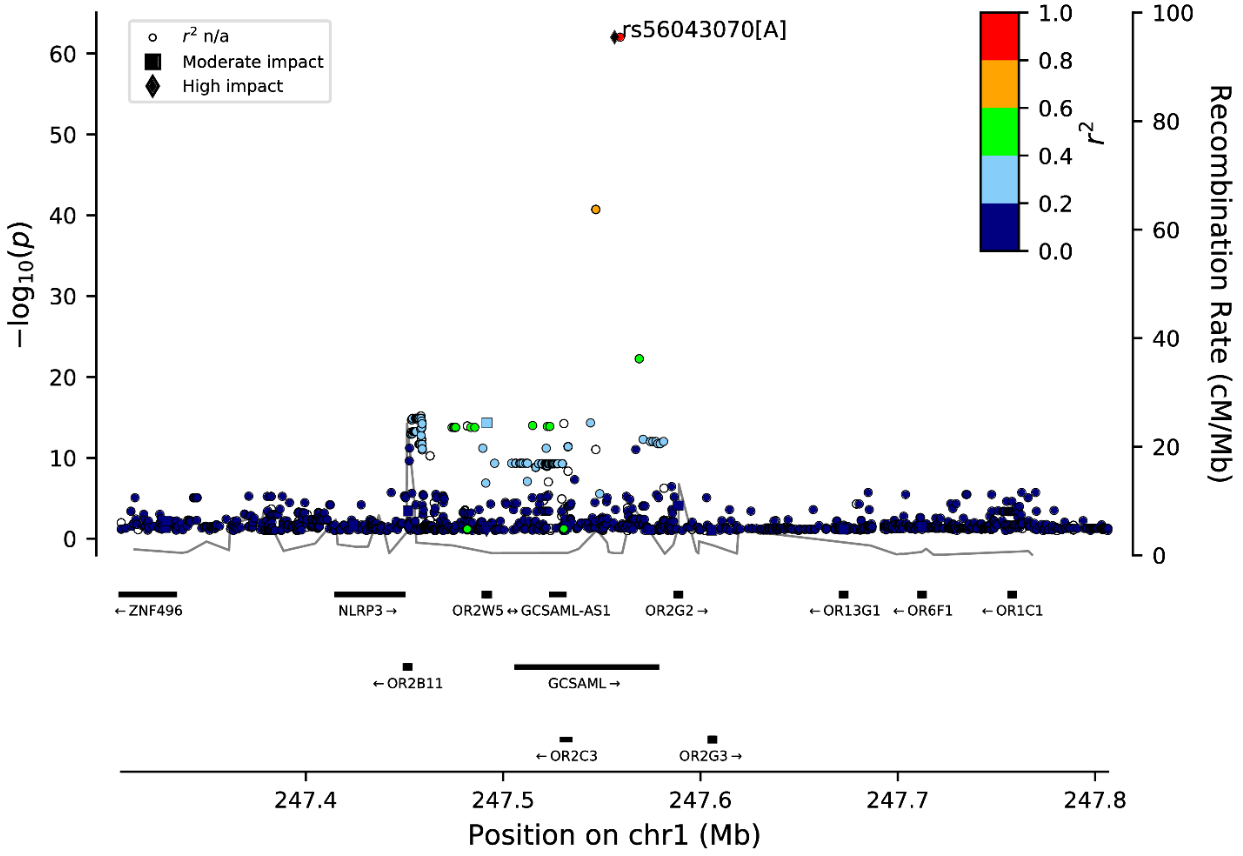

Supplementary Figure 20: Locus plots for the Icelandic data showing the associations of variants at the *GCSAML* locus with RNA-splicing of *GCSAML* in adipose tissue (n = 750). More specifically, variants were associated with percentage spliced-in values (PSI) of the skipping of exon 2 in *GCSAML* using LeafCutter. The leading variant is indicated by its rs number, and other variants are colored according to correlation (r^2) with the leading marker (legend at top-right). Correlation between variants is estimated using genotype data from the Icelandic population. $-\log_{10}P$ values are shown along the left y-axis and correspond to the variants depicted in the plot. The right y-axis shows calculated recombination rates at the chromosomal location, plotted as a solid blue line. The leading variant is strongly correlated with the intronic rs74227709 in *GCSAML* (r^2=1.00, D'=1).

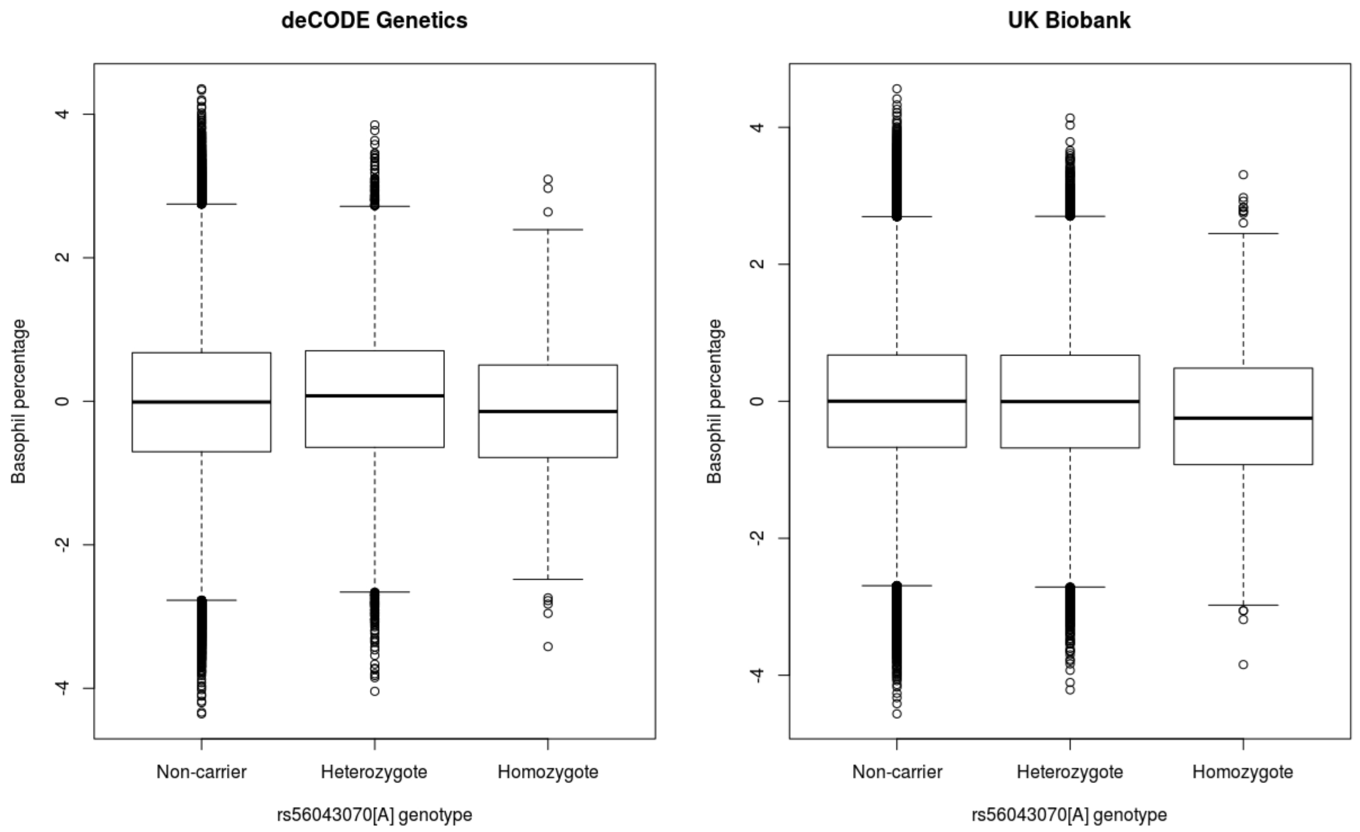

Supplementary Figure 21: Basophil percentages of total white blood cell count in individuals stratified by rs56043070[A] status. The plot on the left depicts data from Icelandic individuals from the dataset at deCODE genetics, while the plot on the right depicts data from white British individuals from the UK Biobank. Basophil percentage is depicted in standardized levels (see Methods).

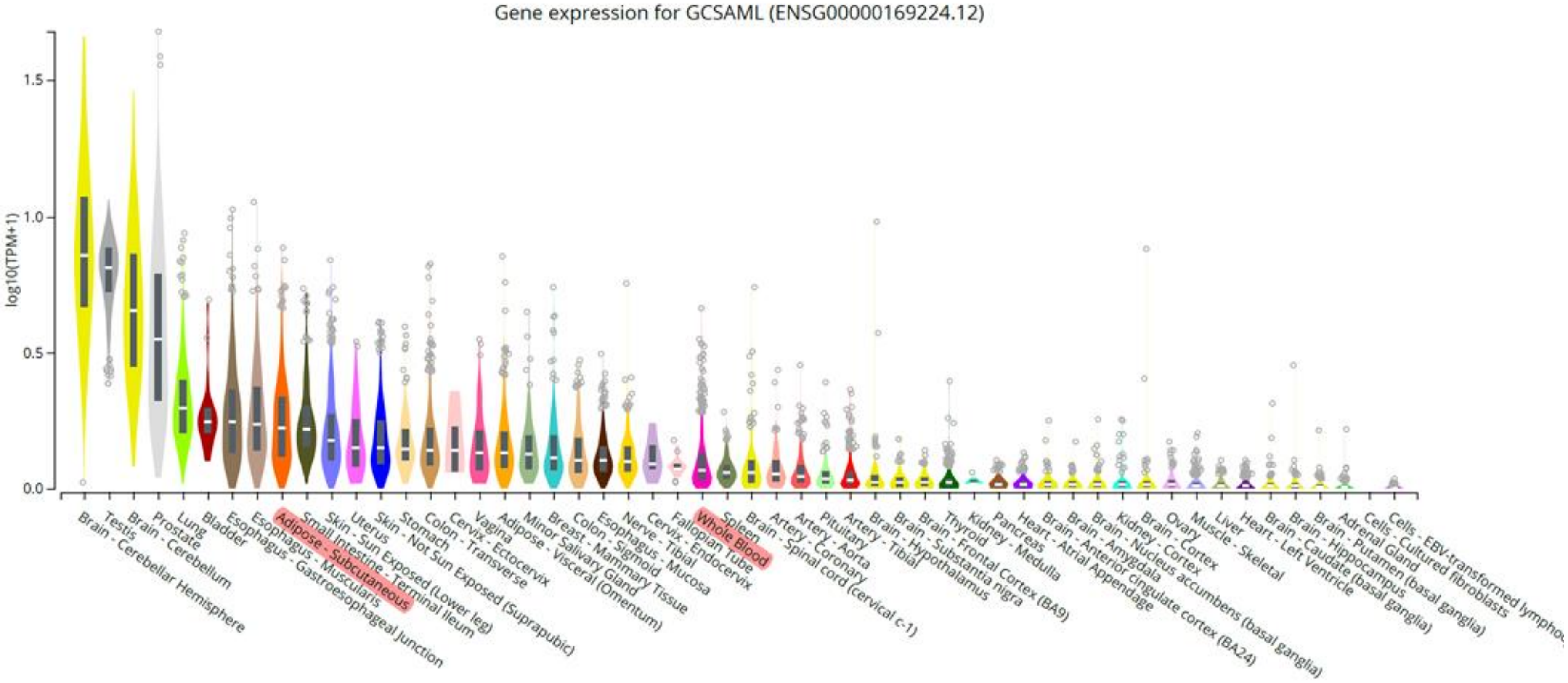

Supplementary Figure 22: Comparison of the distribution of expression levels (in log10(1+TPM)) of *GCSAML* across different tissues in GTEx, with the tissues available in our analysis, subcutaneous adipose tissue and whole blood, highlighted in red.

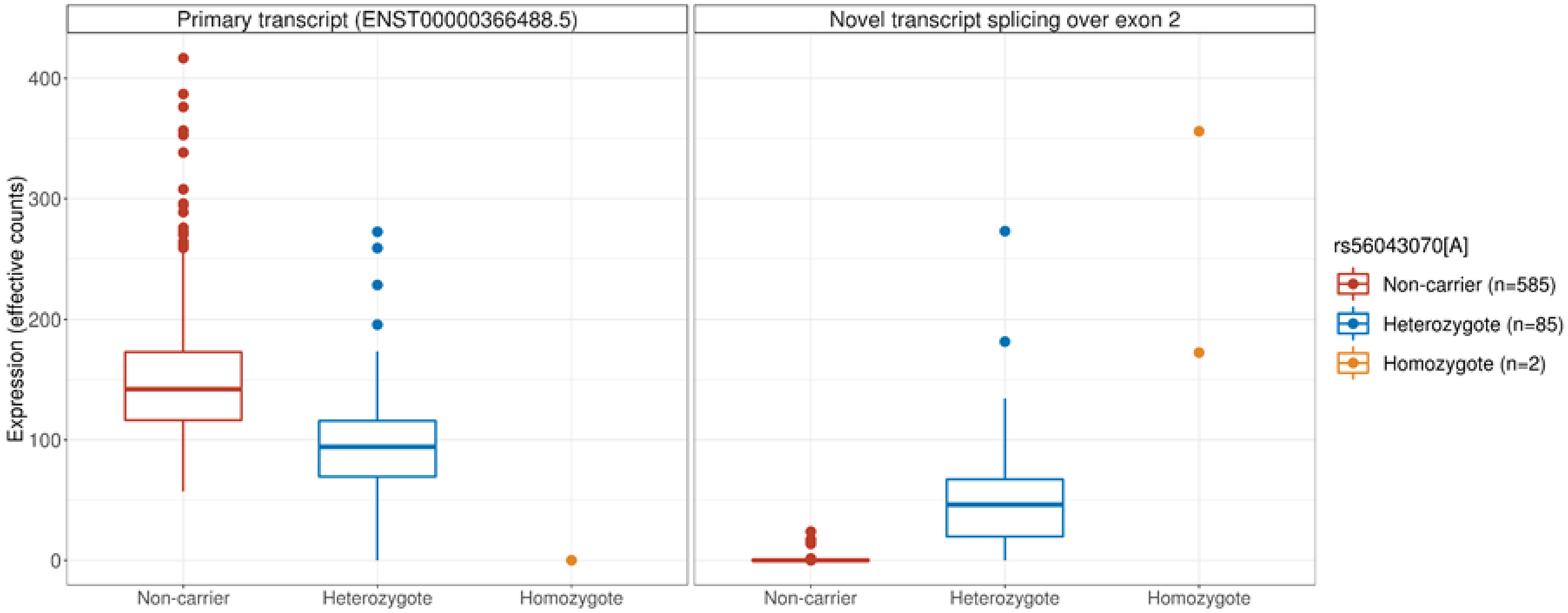

Supplementary Figure 23: Quantification of expression of the three types of *GCSAML* transcripts; The primary transcript (ENST00000366488.5) and novel transcript splicing over exon 2, stratified by rs56043070 genotype. After removal of 78 samples due to lack of expression of the first three exons (see Methods), 672 samples were used for the analysis.

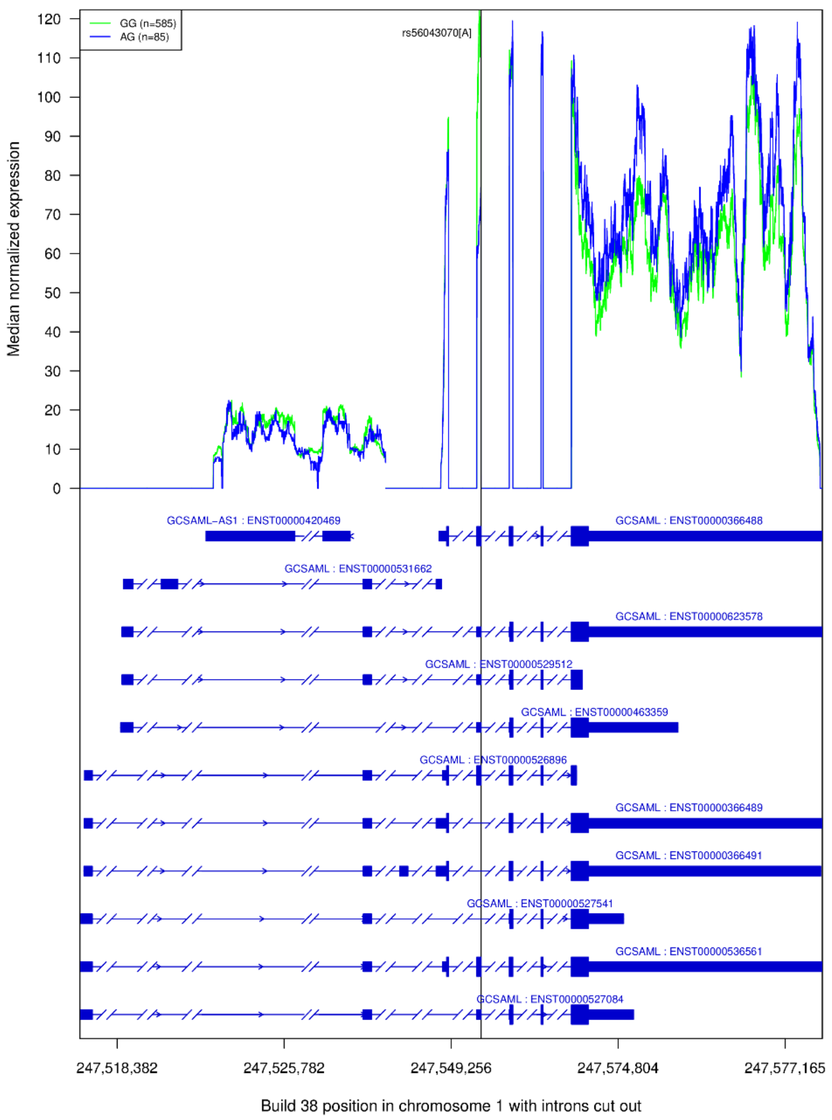

GG (n=585)
AG (n=85)
rs56043070[A]
Median normalized expression
120
110
100
90
80
70
60
50
40
30
20
10
0
GCSAML-AS1 : ENST00000420469
GCSAML : ENST00000366488
GCSAML : ENST00000531662
GCSAML : ENST00000623578
GCSAML : ENST00000529512
GCSAML : ENST00000463359
GCSAML : ENST00000526896
GCSAML : ENST00000366489
GCSAML : ENST00000366491
GCSAML : ENST00000527541
GCSAML : ENST00000536561
GCSAML : ENST00000527084
247,518,382
247,525,782
247,549,256
247,574,804
247,577,165
Build 38 position in chromosome 1 with introns cut out

Supplementary Figure 24: RNA-coverage plot of *GCSAML* in adipose tissue showing the median normalized expression stratified by rs56043070 genotype, with the annotated transcripts in Ensembl v87 shown below. The three transcripts present in Ensembl that splice over exon 2 (enumerated according to the primary transcript ENST00000366488.5), lack median support in their 5' UTR region. In contrast, splicing over exon 2 has substantial support in the STAR alignment data. This motivated further extending the annotation database with an additional transcript identical to the primary transcript except that it splices over exon 2. It turned out that in the quantification step with kallisto and the extended transcriptome, the expression of annotated transcripts splicing over exon 2 is marginal compared to the expression of the novel transcript. The two homozygous carriers of the rs56043070 genotype were left out of the plot for clarity.

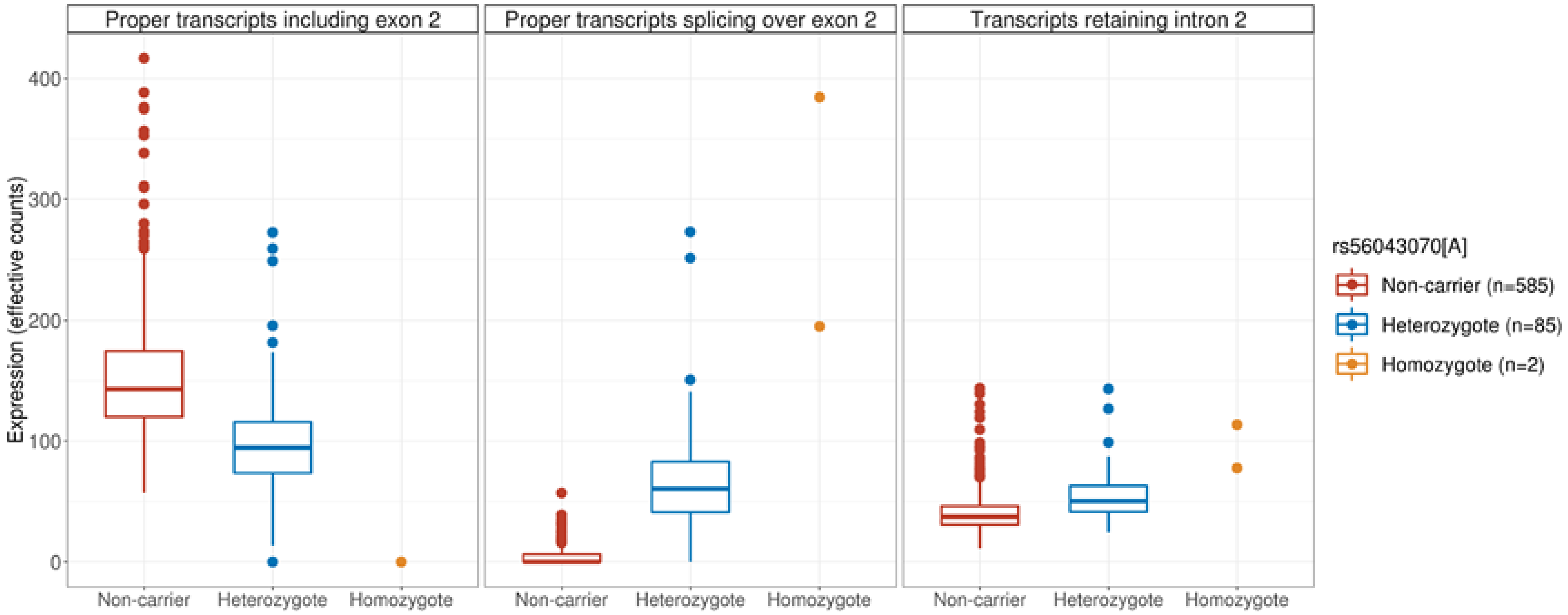

Supplementary Figure 25: Quantification of expression of the three groups of *GCSAML* transcripts; proper transcripts containing exon 2 (including the primary transcript, ENST00000366488.5), proper transcripts splicing over exon 2, and transcripts retaining intron 2, stratified by rs56043070 genotype. After removal of 78 samples due to lack of expression of the first three exons (see Methods), 672 samples were used for the analysis. We use the term proper transcript to refer to transcripts that only use annotated exons and excludes transcripts annotated as having retained introns.

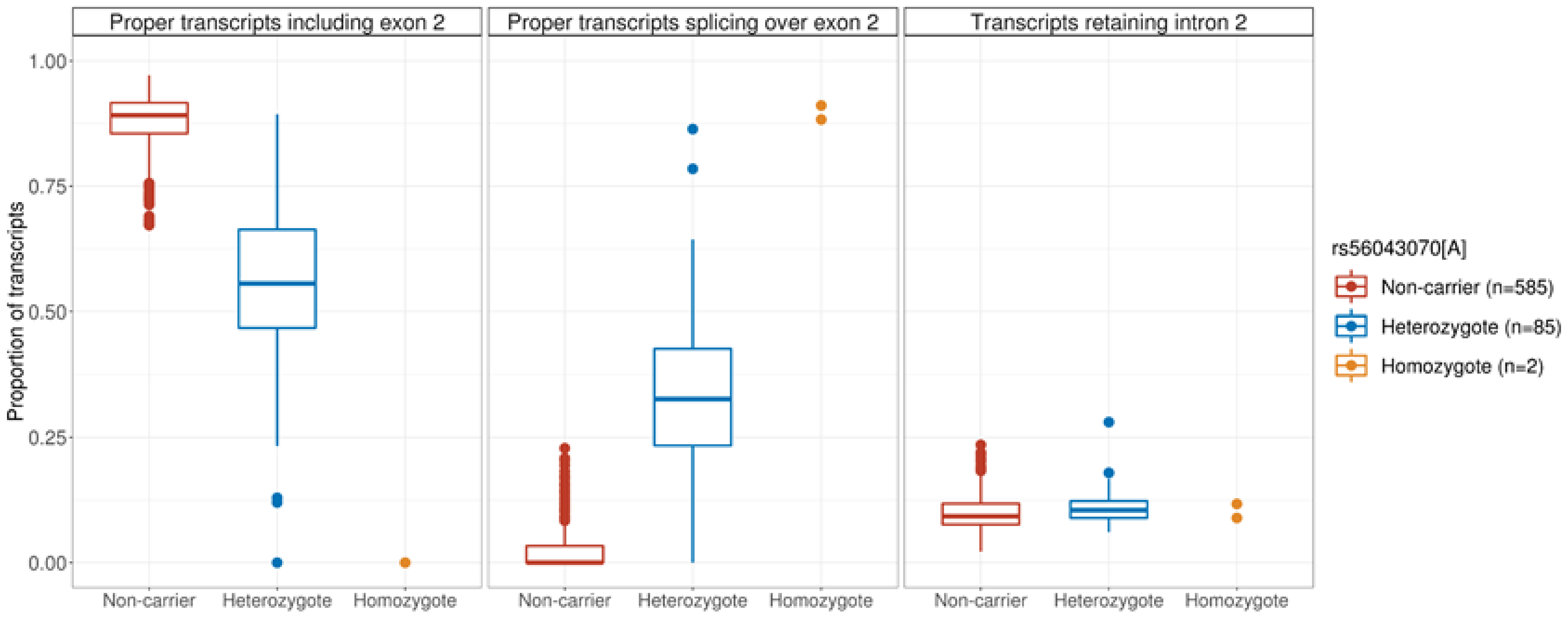

Supplementary Figure 26: Quantification of proportion of the three groups of *GCSAML* transcripts; proper transcripts containing exon 2 (including the primary transcript, ENST00000366488.5), proper transcripts splicing over exon 2, and transcripts retaining intron 2, stratified by rs56043070 genotype. After removal of 78 samples due to lack of expression of the first three exons (see Methods), 672 samples were used for the analysis. We use the term proper transcript to refer to transcripts that only use annotated exons and excludes transcripts annotated as having retained introns.

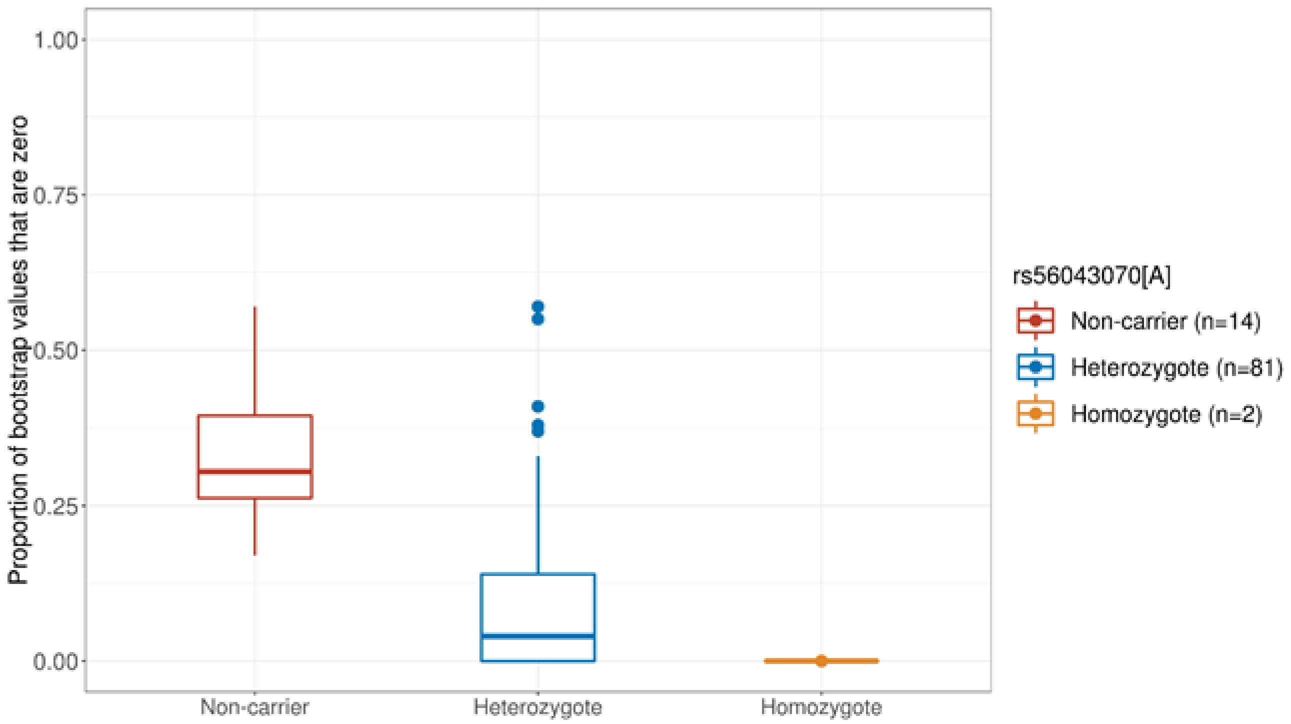

Supplementary Figure 27: Proportion of bootstrap values from kallisto[1] with zero estimated expression of the novel transcript splicing out exon 2 (enumerated according to the primary transcript of *GCSAML*, ENST00000366488.5) stratified by rs56043070 genotype. Only individuals with non-zero expression point estimates of the novel transcript are shown.

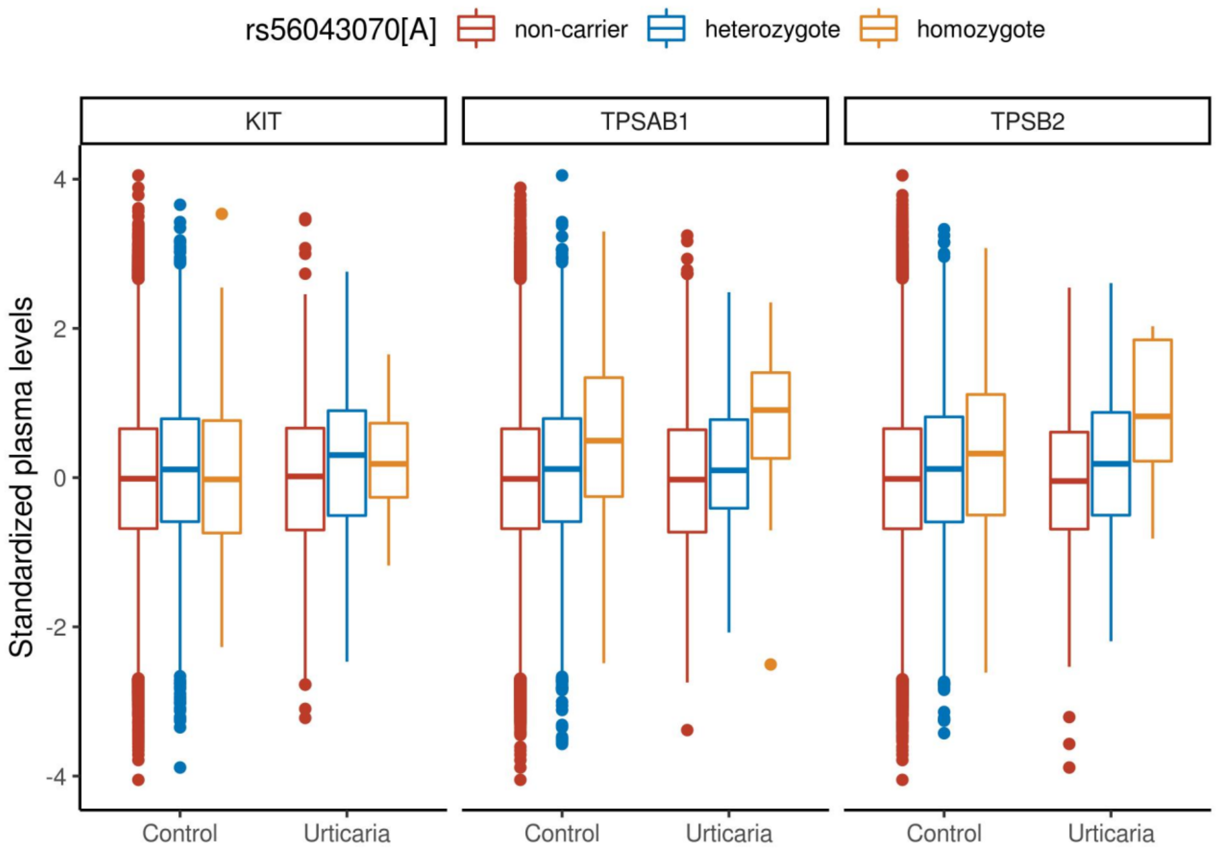

Supplementary Figure 28: The effect of the splice-donor variant rs56043070[A] in *GCSAML* on plasma levels of the protein products of *KIT*, *TPSAB1*, and *TPSB2*, stratified by both genotype ([A] allele carrier status, see legend) and disease-diagnosis status (diagnosed urticaria cases vs. controls). Protein levels are depicted in standardized plasma levels, adjusted for age, sex, and year of sample collection (see Methods).

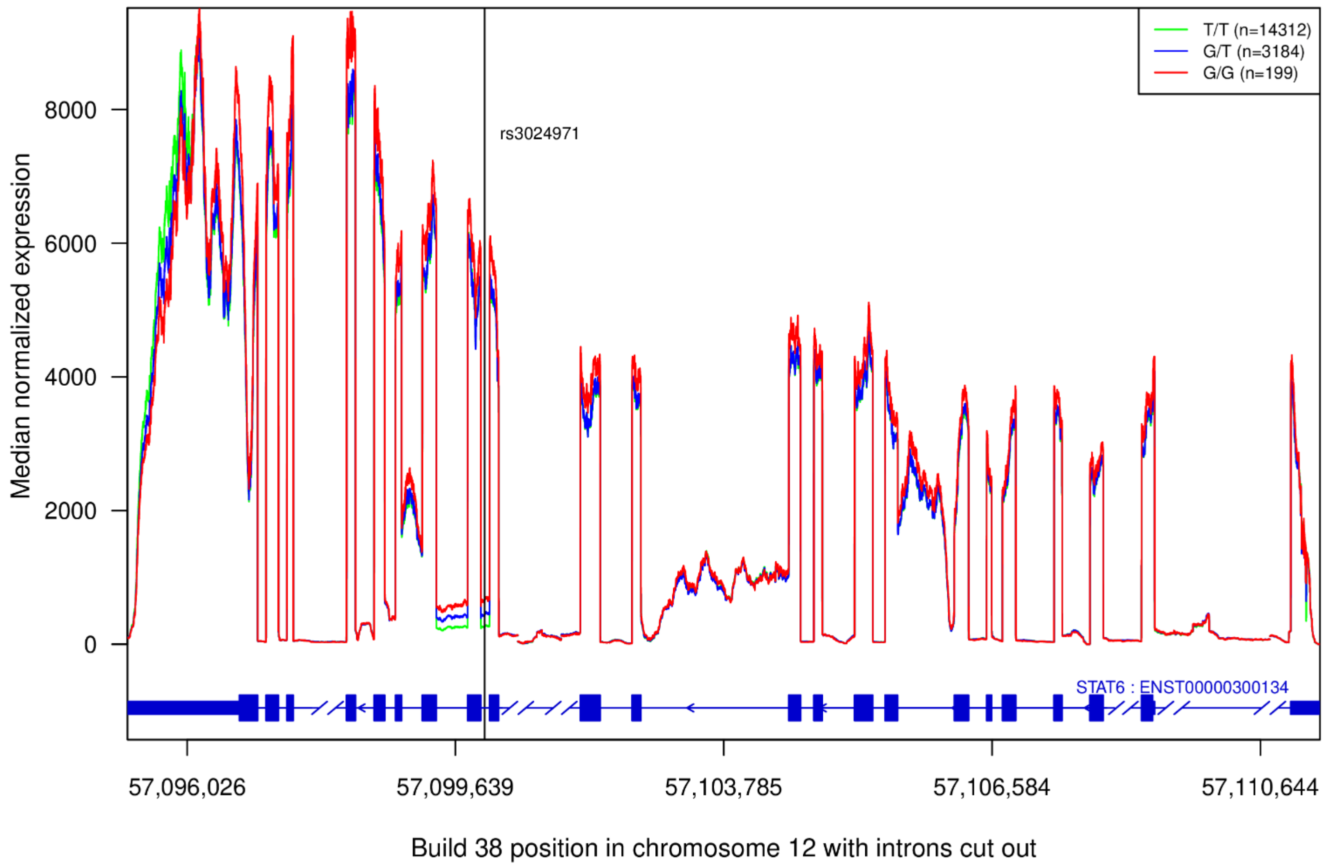

Supplementary Figure 29: RNA-seq coverage plot of *STAT6* from whole-blood, stratified by rs3024971[T] allele. Median coverage of carriers (blue and red) is higher in introns 14 and 15 in concordant with detected sQTL, reported as decreased in splicing between exon 14 to 15 (splice_event_id chr12:57099440:57099766:1378).

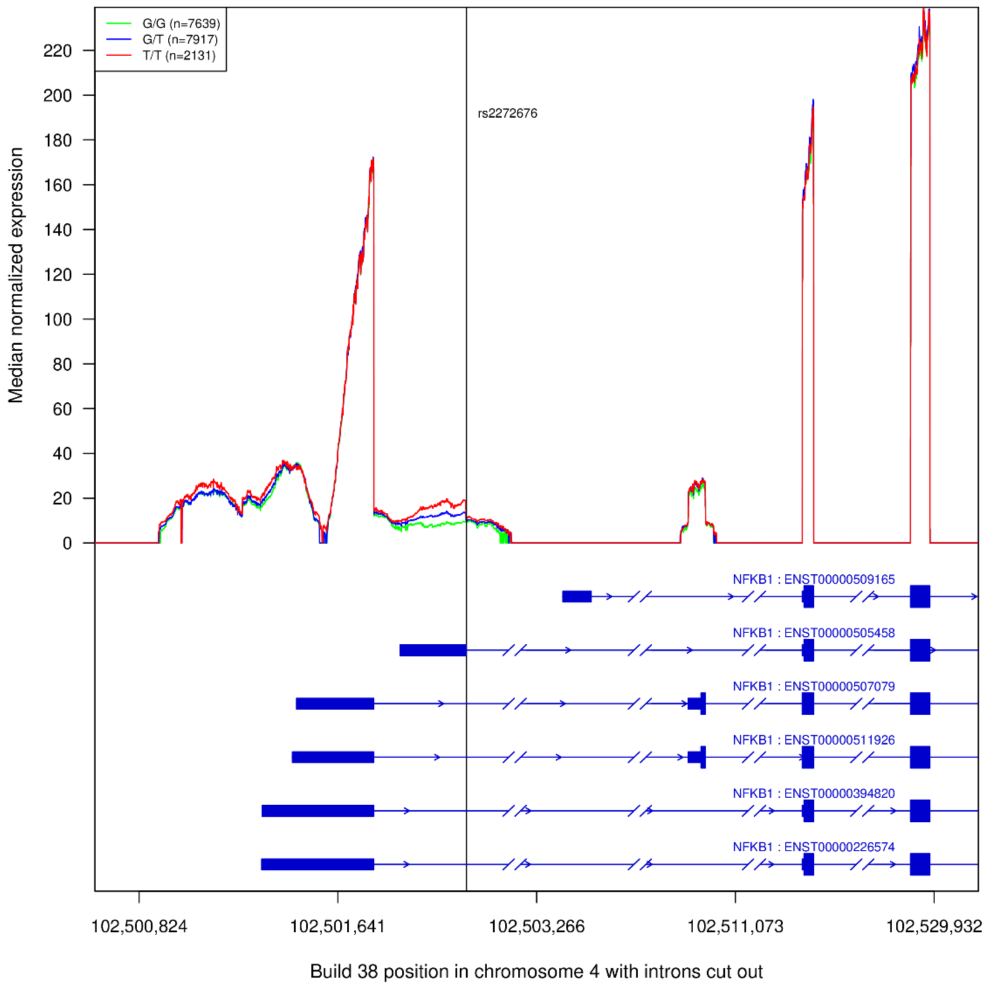

Supplementary Figure 30: RNA sequencing coverage plot of 5‘ region of *NFKB1* from whole-blood, stratified by rs2272676[T] allele. Median coverage of carriers (blue and red) is heigher for the first exon of ENST00000505458 transcript.
