# Supplementary material for: Sequence variant affects GCSAML splicing, mast cell specific proteins, and risk of urticaria
Source: Commun Biol. 2023 Jul 10;6:703. doi: 10.1038/s42003-023-05079-4 (PMC10333346; doi:10.1038/s42003-023-05079-4)
Supplement: Supplementary file 3 — Description of Additional Supplementary Files [file 42003_2023_5079_MOESM3_ESM.pdf]

Description of Additional Supplementary Files

File name: Supplementary Data 1
Description: Associations of sequence variants with urticaria in the meta-analysis

File name: Supplementary Data 2
Description: Associations of sequence variants with urticaria in the meta-analysis under alternative model

File name: Supplementary Data 3
Description: Reported variants and their associations in the meta-analysis for urticaria

File name: Supplementary Data 4
Description: GCSAML transcripts as annotated in the RefSeq database

File name: Supplementary Data 5
Description: Associations of the GCSAML variant with related traits in meta-analyses under alternative model

File name: Supplementary Data 6
Description: Associations of the urticaria associated variants with relevant inflammatory phenotypes in the meta-analyses

File name: Supplementary Data 7
Description: Associations of the urticaria associated variants with quantitative blood traits in meta-analyses

File name: Supplementary Data 8
Description: Reported associations between the urticaria associated variants or highly correlated variants and various phenotypes

File name: Supplementary Data 9
Description: Genetic correlation between Urticaria (ICD 10: L50) and 1,985 ICD10 codes from the UK

File name: Supplementary Data 10
Description: Genetic correlation between Urticaria (ICD 10: L50) and 17 quantitative blood traits from the UK

File name: Supplementary Data 11
Description: Significant associations of the urticaria associated variants with protein levels in plasma in Iceland

File name: Supplementary Data 12
Description: eQTL signals conferred by the urticaria associated variants or highly correlated variants in Icelandic and foreign databases

File name: Supplementary Data 13
Description: Sources for eQTL data

File name: Supplementary Data 14
Description: sQTL signals conferred by the urticaria associated variants or by highly correlated variants

File name: Supplementary Data 15
Description: Correction factors for all tested phenotypes in Iceland and the UK

File name: Supplementary Data 16
Description: Numerical source data for Figure 2b
