# Supplementary material for: Sequence variant affects GCSAML splicing, mast cell specific proteins, and risk of urticaria
Source: Commun Biol. 2023 Jul 10;6:703. doi: 10.1038/s42003-023-05079-4 (PMC10333346; doi:10.1038/s42003-023-05079-4)
Supplement: Supplementary file 5 — Reporting Summary [file 42003_2023_5079_MOESM5_ESM.pdf]

nature portfolio

Corresponding author(s): Patrick Sulem

Last updated by author(s): Jun 12, 2023

Reporting Summary

Nature Portfolio wishes to improve the reproducibility of the work that we publish. This form provides structure for consistency and transparency in reporting. For further information on Nature Portfolio policies, see our Editorial Policies and the Editorial Policy Checklist.

Statistics

For all statistical analyses, confirm that the following items are present in the figure legend, table legend, main text, or Methods section.

n/a	Confirmed	
☐	☒	The exact sample size (*n*) for each experimental group/condition, given as a discrete number and unit of measurement
☐	☒	A statement on whether measurements were taken from distinct samples or whether the same sample was measured repeatedly
☐	☒	The statistical test(s) used AND whether they are one- or two-sided<br>*Only common tests should be described solely by name; describe more complex techniques in the Methods section.*
☐	☒	A description of all covariates tested
☐	☒	A description of any assumptions or corrections, such as tests of normality and adjustment for multiple comparisons
☐	☒	A full description of the statistical parameters including central tendency (e.g. means) or other basic estimates (e.g. regression coefficient) AND variation (e.g. standard deviation) or associated estimates of uncertainty (e.g. confidence intervals)
☐	☒	For null hypothesis testing, the test statistic (e.g. *F*, *t*, *r*) with confidence intervals, effect sizes, degrees of freedom and *P* value noted<br>*Give P values as exact values whenever suitable.*
☒	☐	For Bayesian analysis, information on the choice of priors and Markov chain Monte Carlo settings
☒	☐	For hierarchical and complex designs, identification of the appropriate level for tests and full reporting of outcomes
☐	☒	Estimates of effect sizes (e.g. Cohen's *d*, Pearson's *r*), indicating how they were calculated

Our web collection on statistics for biologists contains articles on many of the points above.

Software and code

Policy information about availability of computer code

Data collection	Not applicable.
Data analysis	We used the following publicly available software for processing of whole-genome sequence data:<br>BWA 0.7.10 mem, https://github.com/lh3/bwa<br>Picard tools 1.117, https://broadinstitute.github.io/picard/<br>SAMtools 1.3, http://samtools.github.io/<br>Bedtools v2.25.0-76-g5e7c696z, https://github.com/arq5x/bedtools2/<br>GraphTyper 1.3, https://github.com/DecodeGenetics/graphtyper<br>Variant Effect Predictor https://github.com/Ensembl/ensembl-vep

For manuscripts utilizing custom algorithms or software that are central to the research but not yet described in published literature, software must be made available to editors and reviewers. We strongly encourage code deposition in a community repository (e.g. GitHub). See the Nature Portfolio guidelines for submitting code & software for further information.

Data

Policy information about availability of data

All manuscripts must include a data availability statement. This statement should provide the following information, where applicable:

- Accession codes, unique identifiers, or web links for publicly available datasets
- A description of any restrictions on data availability
- For clinical datasets or third party data, please ensure that the statement adheres to our policy

GWAS summary statistics will be made available at https://www.decode.com/summarydata/ immediately when the article is published. Sequence variants tested for association have been deposited in the European Variation Archive, accession number PRJEB15197. Other data and code presented in this study are included in this publication, its Supplementary information and upon request.

Research involving human participants, their data, or biological material

Policy information about studies with human participants or human data. See also policy information about sex, gender (identity/presentation), and sexual orientation and race, ethnicity and racism.

Reporting on sex and gender	For all cohorts the results were adjusted for biological sex as a covariate, but no sex-separate analysis was performed.
Reporting on race, ethnicity, or other socially relevant groupings	The study was a meta-analysis of GWAS for urticaria using data from Iceland, the UK, Finland and Japan. In GWAS we want each cohort to be as genetically homogeneous as possible to avoid confounders as much as possible. Closer relatedness and kinship increase homogeneity. The cohorts represent the populations they belong to. However in UK it is specified that only individuals of European descent were used in the study.
Population characteristics	A detailed description of population characteristics can be found in the methods section. In Iceland, sex, county of birth, current age or age at death (first and second order terms included), blood sample availability for the individual and an indicator function for the overlap of the lifetime of the individual with the time span of phenotype collection were included as covariates in the logistic regression model. For the UK, sex, age, and the first 40 principal components were included as covariates. The Finngen association analysis for binary traits was adjusted for sex, age, the genotyping batch, and the first 10 principal components. In the association analysis from Biobank Japan, both binary and quantitative traits were adjusted for age, sex, and the top 20 principal components as covariates.
Recruitment	For the deCODE Genetics study individuals were recruited through various research projects at deCODE genetics. The participants are a large fraction of the adult Icelandic population.<br>UK Biobank holds data on half a million participants throughout the UK. All participants in UK Biobank were recruited through assessment centres, designed specifically for this purpose. The FinnGen database consists of samples collected from Finnish biobanks. The BioBank Japan database consists of samples collected from Japanese biobanks.
Ethics oversight	All participating subjects in the deCODE genetics study who donated blood signed informed consent. The personal identities of the participants and biological samples were encrypted by a third-party system. The study was approved by the Icelandic Data Protection Authority and the National Bioethics Committee of Iceland (no VSN-14-099).<br>The FinnGen database consists of samples collected from the Finnish biobanks and and phenotype data collected at the national health registers. The Coordinating Ethics Committee of the Helsinki and Uusimaa Hospital District evaluated and approved the FinnGen research project. The project complies with existing legislation (in particular the Biobank Law and the Personal Data Act). The official data controller of the study is University of Helsinki.<br>The UK Biobank Resource was used under application number 56270. All phenotype and genotype data were collected following an informed consent obtained from all participants. The North West Research Ethics Committee reviewed and approved UK Biobank's scientific protocol and operational procedures (REC Reference Number: 06/MRE08/65).<br>Biobank Japan collected phenotype data (medical history and clinical data) and serum samples at participating medical institutions in Japan. All participants provided written, informed consent and the study was approved by ethics committees of the Institute of Medical Sciences, the University of Tokyo and RIKEN Center for Integrative Medical Sciences.

Note that full information on the approval of the study protocol must also be provided in the manuscript.

Field-specific reporting

Please select the one below that is the best fit for your research. If you are not sure, read the appropriate sections before making your selection.

☒ Life sciences ☐ Behavioural & social sciences ☐ Ecological, evolutionary & environmental sciences

For a reference copy of the document with all sections, see nature.com/documents/nr-reporting-summary-flat.pdf

Life sciences study design

All studies must disclose on these points even when the disclosure is negative.

Sample size	We combined 14,312 cases and 354,647 controls from Iceland, 8,730 cases and 399,923 controls from the UK, 7,759 cases and 313,241 controls from Finland, 9,893 cases and 162,190 controls from Japan; in total, 40,694 cases and 1,230,001 controls and correspond to all available data

Data exclusions	No available data was excluded from the study
Replication	No replication performed
Randomization	Not applicable (GWAS study, not a randomized trial)
Blinding	Blinding Not applicable (GWAS study, not a randomized trial, so no blinding is required)

Reporting for specific materials, systems and methods

We require information from authors about some types of materials, experimental systems and methods used in many studies. Here, indicate whether each material, system or method listed is relevant to your study. If you are not sure if a list item applies to your research, read the appropriate section before selecting a response.

Materials & experimental systems

n/a	Involved in the study
☒	☐ Antibodies
☒	☐ Eukaryotic cell lines
☒	☐ Palaeontology and archaeology
☒	☐ Animals and other organisms
☒	☐ Clinical data
☒	☐ Dual use research of concern
☒	☐ Plants

Methods

n/a	Involved in the study
☒	☐ ChIP-seq
☒	☐ Flow cytometry
☒	☐ MRI-based neuroimaging
